# Bridge Functionalisation of Bicyclo[1.1.1]pentane Derivatives

**DOI:** 10.1002/anie.202106352

**Published:** 2021-08-07

**Authors:** Joseph M. Anderson, Nicholas D. Measom, John A. Murphy, Darren L. Poole

**Affiliations:** ^1^ GlaxoSmithKline Medicines Research Centre Gunnels Wood Road Stevenage Hertfordshire SG1 2NY UK; ^2^ Department of Pure and Applied Chemistry WestCHEM University of Strathclyde 295 Cathedral Street Glasgow Scotland G1 1XL UK

**Keywords:** bicyclopentanes, bioisosteres, propellanes, radical reactions, small-ring systems

## Abstract

“Escaping from flatland”, by increasing the saturation level and three‐dimensionality of drug‐like compounds, can enhance their potency, selectivity and pharmacokinetic profile. One approach that has attracted considerable recent attention is the bioisosteric replacement of aromatic rings, internal alkynes and *tert*‐butyl groups with bicyclo[1.1.1]pentane (BCP) units. While functionalisation of the tertiary bridgehead positions of BCP derivatives is well‐documented, functionalisation of the three concyclic secondary bridge positions remains an emerging field. The unique properties of the BCP core present considerable synthetic challenges to the development of such transformations. However, the bridge positions provide novel vectors for drug discovery and applications in materials science, providing entry to novel chemical and intellectual property space. This Minireview aims to consolidate the major advances in the field, serving as a useful reference to guide further work that is expected in the coming years.

## Graphical abstract

This Minireview summarises the known chemistry for the preparation of bicyclo[1.1.1]pentane (BCP) derivatives reported up to mid‐2021. Bridge substitution on BCP derivatives often provides patent‐free, novel vectors for substituent disposition in drug discovery and materials science; this capacity may prove to be transformational in the coming years.

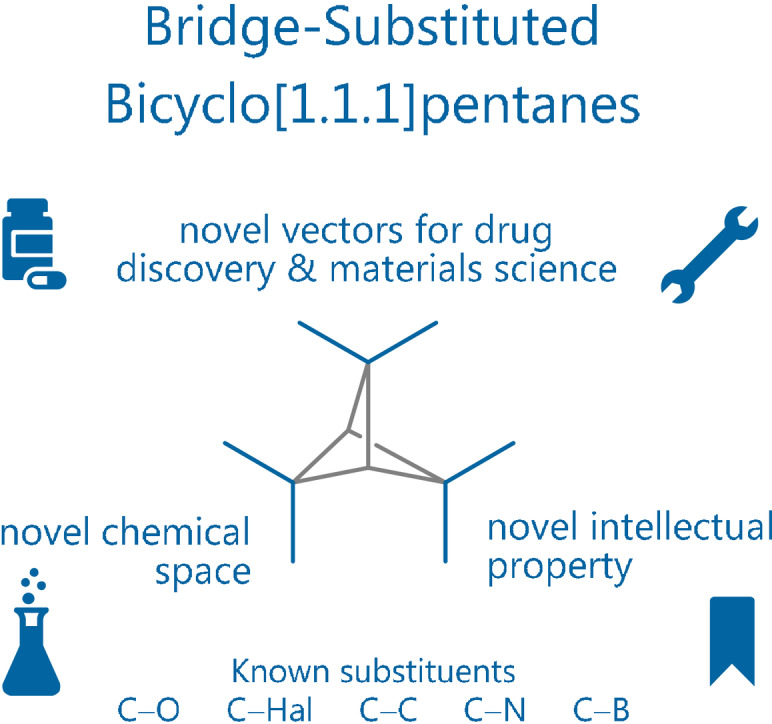

## 1 Introduction

Over the past three decades, the application of bicyclo[1.1.1]pentane (BCP) derivatives in materials science as molecular rods,^[1]^ molecular rotors,^[2]^ supramolecular linker units,^[3]^ liquid crystals,^[4]^ FRET sensors^[5]^ and metal–organic frameworks^[6]^ has been extensively investigated and is the subject of a recent review.^[7]^ Since the seminal reports of Barbachyn (1993)^[8]^ and Pellicciari (1996),^[9]^ the BCP motif has also emerged within drug discovery as a valuable bioisostere for internal alkynes, *tert*‐butyl groups and monosubstituted/1,4‐disubstituted arenes.^[10]^ The appeal of the abiotic BCP fragment, among related systems such as cubanes and higher bicycloalkanes, originates from its ability to add three‐dimensional character and saturation to compounds. Increasing the fraction of *sp* ^3^‐hybridised carbon atoms in a drug molecule, F*sp* ^3^, has been found to make a lead oral drug compound “more developable” ^[11]^ and correlates positively with clinical success.^[12]^ To this end, multiple research groups have documented the increased or equal solubility^[13]^/potency[^8^, ^9^, ^13a^, ^13d^, ^14^]/metabolic stability[^13a^, ^13c^, ^13e^, ^15^] and decreased non‐specific binding[^13c^, ^13f^] of lead compounds that can be achieved through such bioisosteric replacements. Increasing the solubility and potency of a medicine can reduce the therapeutic dose required, potentially avoiding drug–drug interactions and drug‐induced liver injury through metabolic activation. Bioisosteric replacement has also been identified as a strategy to circumvent Markush structure patent claims on drug candidates.^[16]^

**Scheme 1:**
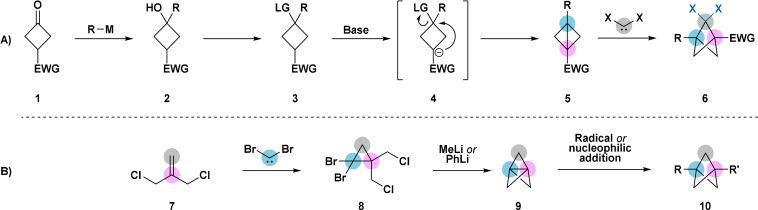
A) Synthesis of the bicyclo[1.1.1]pentane framework through carbene insertion into the central bond of a bicyclo[1.1.0]butane. B) Synthesis of the bicyclo[1.1.1]pentane framework via radical or nucleophilic addition across a [1.1.1]propellane. LG=leaving group. EWG=electron‐withdrawing group. X=F, Cl, Br.

Multiple reviews have addressed the 12+ unique synthetic approaches that have been reported for construction of the BCP framework.[^7^, ^10^, ^17^] In particular, carbene insertion into the central bond of bicyclo[1.1.0]butanes, and nucleophilic/radical addition across the central bond of [1.1.1]propellanes, have emerged as the two most practical and scalable methods (Scheme 1). Methodology now exists to install most fragments of interest in drug discovery at the bridgehead (1,3) positions. In contrast, methodology for substitution of the bridge (2,4,5) positions remains underdeveloped.^[18]^ Most reported methods forge the key substituent bond prior to construction of the BCP core itself. For reasons outlined in Section 2.2, examples of the direct functionalisation of the bridge positions in a controlled manner are rare.

Bridge‐substituted BCP derivatives represent novel chemical space and provide novel vectors for ligand–target interactions in drug discovery. As successful drug discovery becomes more challenging year on year,^[19]^ accessing new chemical space becomes increasingly important. Introduction of the abiotic BCP motif may also provide a new strategy for overcoming genetic resistance to existing therapeutic compounds. Brown has recently commented on this issue,^[20]^ highlighting that abiotic scaffolds may prove important in the discovery of anticancer, antibiotic, antiviral and antiparasitic drugs, where the causative agents can be highly proficient at developing resistance to established treatments.

Aspects of the bridge functionalisation of BCP derivatives have been previously addressed by Michl and co‐workers.^[17a]^ Herein, we seek to update this work and provide a summary, from a synthetic perspective, of the known methods for the preparation of bridge‐substituted BCP derivatives reported up to mid‐2021. The discussion is organized by the nature of the substituent installed. Further distinction is drawn between strategies that install the substituent before synthesis of the BCP core, and those proceeding via direct functionalisation of the hydrocarbon skeleton.

## 2 Structural Chemistry of the Bicyclo[1.1.1]pentane Core

### 2.1 General Considerations

The BCP core is highly strained yet remarkably stable, with a strain energy of 65^[17a]^–68^[21]^ kcal mol^−1^ for the parent hydrocarbon (cf. 27.5 kcal mol^−1^ for cyclopropane^[21]^) and thermal stability up to ca. 300 °C.[^17a^, ^22^] This strain primarily arises from significant destabilizing overlap of the rear lobes of bridgehead orbitals directed at substituent positions.^[23]^ In many cases, this transannular communication attenuates and directs the reactivity of the cage.

### 2.2 Orbital Hybridisation and C−H Bond Strengths in the BCP Core

The hybridisations of bridge and bridgehead carbon orbitals used for substituent bonding on the parent hydrocarbon have been established as *sp* ^2.5^ and *sp* ^2.0^ respectively through ^1^ *J*_C‐H_ coupling constant analysis,^[24]^ in good agreement with values obtained from ab initio^[25]^ and semiempirical^[26]^ calculations. The high *s* character of these orbitals has two effects: 1) bicyclopent‐1‐yl and bicyclopent‐2‐yl fragments are net electron‐withdrawing;^[17a]^ and 2) BCP C−H bonds have a large homolytic bond dissociation energy (BDE), greater than that of typical saturated hydrocarbons. Indeed, Maillard and Walton have demonstrated that hydrogen atom abstraction by *tert*‐butoxyl radicals proceeds more rapidly from cyclopropane than from the parent BCP.^[27]^

Furthermore, although simple consideration of hybridisation suggests that the bridge C−H bonds should be weaker than bridgehead C−H bonds, *only* the bridgehead radical is observed by EPR on exposure of BCP to *tert*‐butoxyl radicals. This regioselectivity is remarkable considering that the bridge and bridgehead radicals have approximately equal calculated enthalpies of formation,^[28]^ and that the bridge hydrogens have a threefold statistical advantage over the bridgehead hydrogens. Aside from the bridgehead C−H bonds being more sterically accessible,^[29]^ the selectivity arises from the aforementioned transannular interactions within the BCP core. The transition state for hydrogen atom abstraction by an electronegative radical is polarised, with development of partial positive charge on the adjacent carbon. During hydrogen abstraction from a bridgehead site, this positive charge can be extensively stabilised by the transannular bridgehead orbital interaction.^[17a]^ In contrast, the bridge carbon does not have access to such stabilisation and so hydrogen atom transfer (HAT) from this position is kinetically inhibited. Moreover, if one bridgehead position is functionalised with an electron‐withdrawing group, this intracage stabilisation is compromised and so the expected selectivity of C–H abstraction from a bridge position is returned (see Section 4.1).^[30]^

In summary, the high BDE of BCP C−H bonds means that a direct HAT approach for decoration of bridge positions would likely be confined to a very limited range of substrates; they could possess no other alkyl C−H bonds, and blocking of unfunctionalised bridgehead positions may be necessary. More recently, attempts at *directed* C–H activation chemistry on the bridge positions have also proven unsuccessful.^[31]^ Consequently, prefunctionalisation at the bridge positions is expected to be necessary for the divergent introduction of substituents.

### 2.3 Reactive Intermediates on BCP Bridge Positions

The generation and properties of reactive intermediates (carbocations, carbanions and radicals) on the bridgehead and bridge positions of BCP derivatives have been extensively discussed by Michl.^[17a]^ Key points relevant to the synthesis of bridge‐functionalised derivatives are:

1. Bridge‐centered carbenium ions are unstable, undergoing rapid and irreversible skeletal rearrangement to ring‐opened products (see Sections 3.1, 4.1 and 5.2). As such, they are not viable intermediates for the preparation of bridge‐substituted BCP derivatives.
2. Bridge‐centered carbanions are kinetically stable against ring opening, and are putative intermediates in the Haller–Bauer cleavage and Birch reduction of 2‐substituted BCP derivatives (see Section 5.2).
3. Bridge carbon‐centered radicals are kinetically stable against β‐scission to afford cyclobutyl products, even though this process is calculated to be strongly exothermic.^[32]^

## 3 Oxygen Substituents

### 3.1 Bicyclo[1.1.1]pentan‐2‐ol and Derivatives

**Scheme 2:**
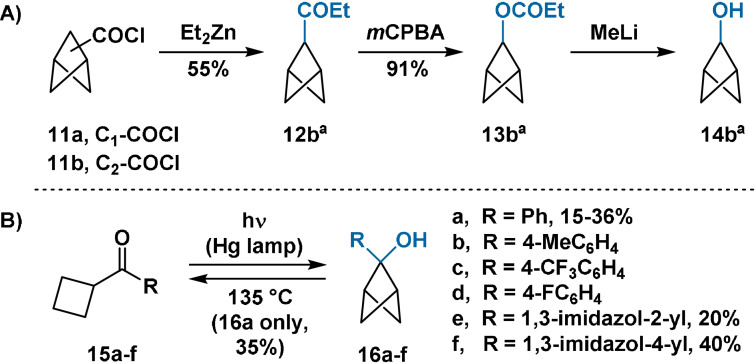
A) Synthesis of bicyclo[1.1.1]pentan‐2‐ol (**14 b)** from acyl chloride **11 b**. B) Synthesis of alcohols **16 a**–**f** through NY cyclisation. *m*CPBA=*meta*‐chloroperoxybenzoic acid. **16 b**–**d**; chemical yields not reported. [a] Obtained as a mixture with the corresponding bridgehead‐substituted compound.

Methods for the preparation of BCP bridge alcohols are rare. Attempts at direct C–H oxidation using persulfate salts have been unsuccessful.^[30]^ The parent bicyclo[1.1.1]pentan‐2‐ol (**14 b**) has been most efficiently accessed through Baeyer–Villiger oxidation of mixed ketones **12** (see Section 5.1), hydrolysis of the resulting esters and separation of the isomeric alcohols (Scheme 2 A).^[29]^ Compound **14 b** has also been accessed in low yield through reduction of the corresponding ketone (see Section 3.2). The 2‐substituted phenyl analogue (**16 a**) is more accessible,^[33]^ representing the major product of the photolysis of cyclobutyl phenyl ketone via (thermally reversible)[^33a^, ^33b^] Norrish–Yang (NY)^[34]^ cyclisation (Scheme 2 B). The NY cyclisation strategy is compatible with electron‐poor and electron‐neutral substrates (**16 b**–**f**), but is less tolerant of electron‐rich systems such as vinyl and 2‐furyl cyclobutyl ketones.^[35]^

**Scheme 3:**
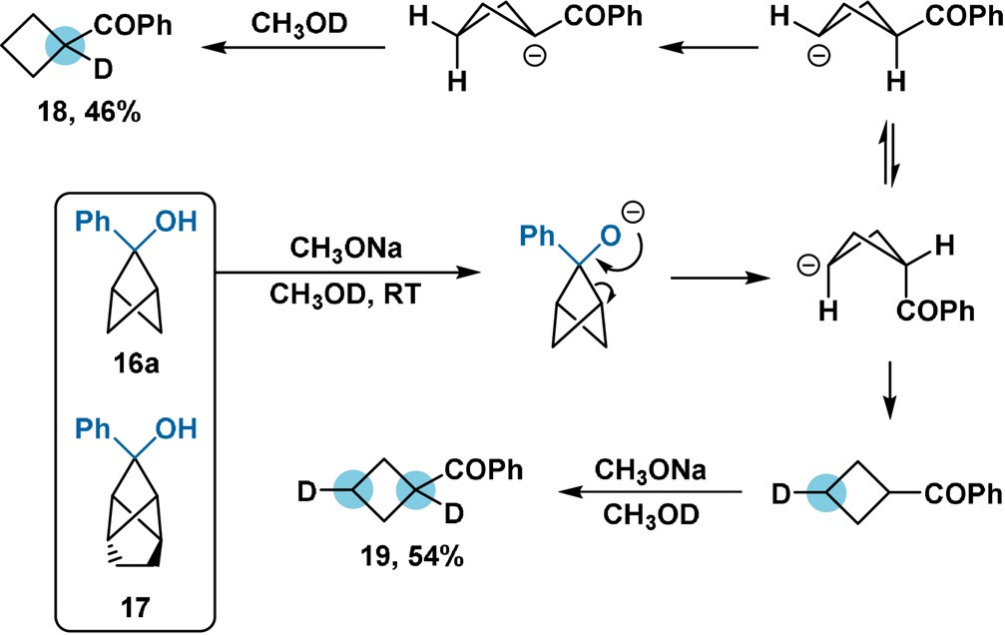
Deuterium‐labelling studies on alcohol **16 a**, identifying two competitive mechanisms for ring opening. The structure of tricyclic alcohol **17**.

While the lithium salt of the parent bicyclo[1.1.1]pentan‐2‐ol is stable in aprotic solvents (Scheme 2 A),^[29]^ increased strain at the bridge position means that 2‐substituted bicyclo[1.1.1]pentan‐2‐ol alkoxides are unstable. Exposure of alcohols **16 a** and **17** to catalytic NaOMe in methanol results in rapid cycloreversion to the corresponding cyclobutyl phenyl ketones,[^33b^, ^36^] analogously to the ring‐opening of cyclopropyl alcohols.^[37]^ Deuterium‐labelling studies have identified two distinct pathways for ring opening (Scheme 3); however, both are driven by release of ring strain in the transition state.^[36a]^ Ring opening occurs rapidly at cryogenic temperatures; attempts at *O*‐alkylation of the alkoxide of **16 a** with methyl iodide or dimethyl sulfate have been unsuccessful.^[36a]^ Alkylation is presumably retarded by the decreased nucleophilicity of the alkoxide from the electron‐withdrawing effect and steric demand of the BCP moiety.

**Scheme 4:**
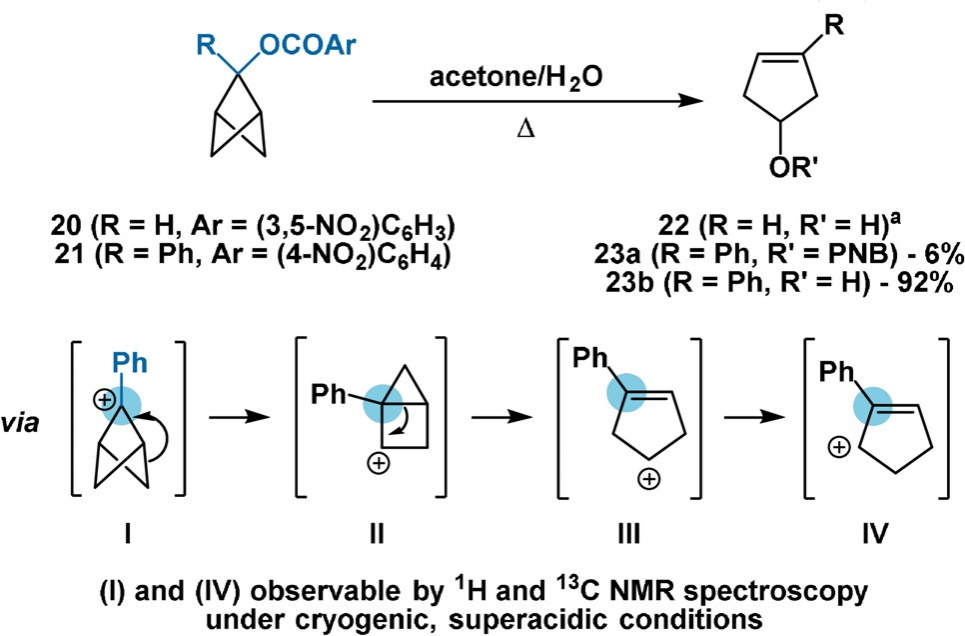
Solvolysis of aryl esters **20** and **21**. PNB=*para*‐nitrobenzoyl. [a] Sole product; yield not reported.

Moreover, the *p*‐bromophenylcarbamate derivative of alcohol **16 a** has been prepared through direct reaction with the isocyanate without prior deprotonation.^[33c]^ Similarly, nitrobenzoyl esters **20** ^[38]^ and **21**[^36a^, ^39^] have been prepared by treatment of alcohols **14 b** and **16 a**, respectively, with the appropriate benzoyl chloride. Solvolysis of these esters leads only to ring‐opened products, illustrating the instability of BCP‐centered carbocations. Similarly, treatment of free alcohol **16 a** with acetic acid^[36a]^ produces **22** in 40 % yield and the corresponding acetate in 60 % yield. The mechanism of rearrangement has been studied kinetically and spectroscopically.[^36a^, ^38^, ^39^, ^40^] It is proposed that departure of the leaving group is assisted by participation of an adjacent C−C σ‐bond (Scheme 4). Rearrangement, presumably via housane cation **II**, provides cyclopentenyl cation **III** which may be trapped by nucleophiles. While cation **I** has been observed by NMR spectroscopy under cryogenic, superacidic conditions, cation **II** was not detected. In the absence of nucleophiles, **III** is observed as allylic cation **IV** arising from formal Wagner–Meerwein rearrangement.^[40]^

**Scheme 5:**
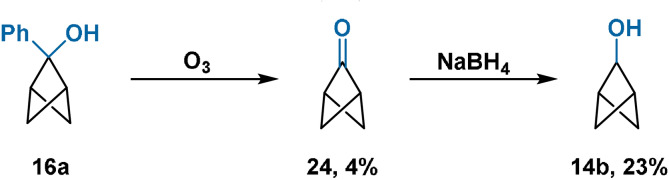
Synthesis of ketone **24** through ozonolysis of **16 a**, and subsequent NaBH_4_ reduction affording **14 b**.

In summary, access to the parent bicyclo[1.1.1]pentan‐2‐ol (**14 b**) remains extremely inefficient (Schemes 2 and 5). Additionally, the instability of **14 b** and derivatives makes them challenging synthetic intermediates to handle, unless they can be protected as solvolytically stable functionalities under strictly neutral conditions.

### 3.2 Bicyclo[1.1.1]pentan‐2‐one and Derivatives

Ketone **24** has been prepared by Dougherty through ozonolysis of alcohol **16 a**.^[42]^ NaBH_4_ reduction then provided alcohol **14 b** in 23 % yield (Scheme 5). The poor yield of the ozonolysis reaction precludes **24** as a useful synthetic intermediate for the preparation of 2‐substituted BCP derivatives. Compound **24** is thermally unstable, undergoing cycloreversion at elevated temperatures to afford allylketene and derived oligomers/nucleophilic trapping products. Cycloreversion is much more facile than for typical cyclobutanones. Ketalisation of **24** has only been achieved through irradiation in CD_3_OD in the absence of base.^[42]^

**Scheme 6:**
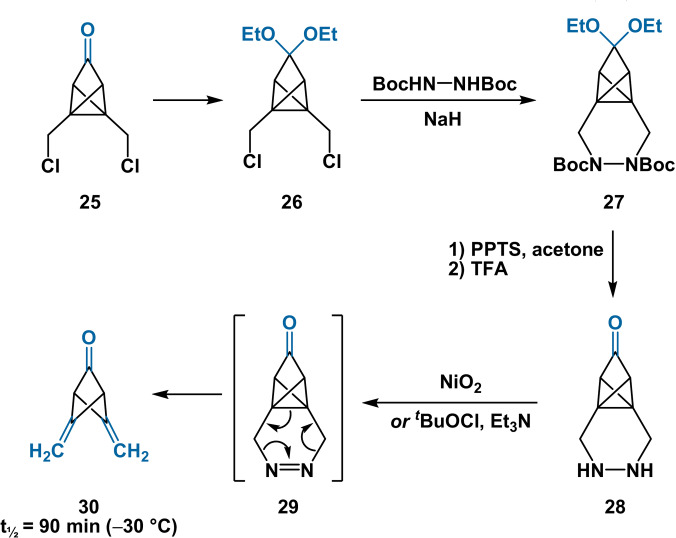
Synthesis of 4,5‐dimethylenebicyclo[1.1.1]pentan‐2‐one (**30**). Boc=*tert*‐butoxycarbonyl; PPTS=pyridinium *p*‐toluenesulfonate; TFA=trifluoroacetic acid; *t*_1/2_=half‐life.

The related 4,5‐dimethylenebicyclo[1.1.1]pentan‐2‐one (**30**) was later prepared by Dowd from tricyclopentane **25**,^[41]^ where generation of the highly strained BCP scaffold is compensated by extrusion of dinitrogen from unstable intermediate **29** (Scheme 6). The instability of **30** similarly precludes its use as a synthetic intermediate to access bridge‐substituted BCP derivatives.

## 4 Halogen Substituents

Only bridge‐fluorinated, ‐chlorinated and ‐brominated BCP derivatives are known. While fluorine and chlorine substituents have been introduced through both dihalocarbene insertion into the central bond of bicyclo[1.1.0]butane precursors and through direct reaction of BCP derivatives with the elemental halogens, bromine has only been introduced through dibromocarbene insertion.[^30^, ^43^]

### 4.1 Bridge‐Chlorinated derivatives

The dichlorocarbene insertion method of Cherkofsky^[43a]^ (Scheme 1) is arguably the most convenient approach to bridge chlorine‐substituted BCP derivatives. Both chloroform^[43a]^ and sodium trichloroacetate^[13d]^ are viable carbene precursors. Monodechlorination is readily achieved using tin^[30]^ or silicon^[13d]^ hydride radical chemistry, facilitated by stabilisation of the transition state for chlorine abstraction by the remaining halogen atom. Removal of the second halogen atom, to afford a bridge‐unfunctionalised BCP derivative, is possible but requires forcing reaction conditions and extended reaction times.

**Scheme 7:**
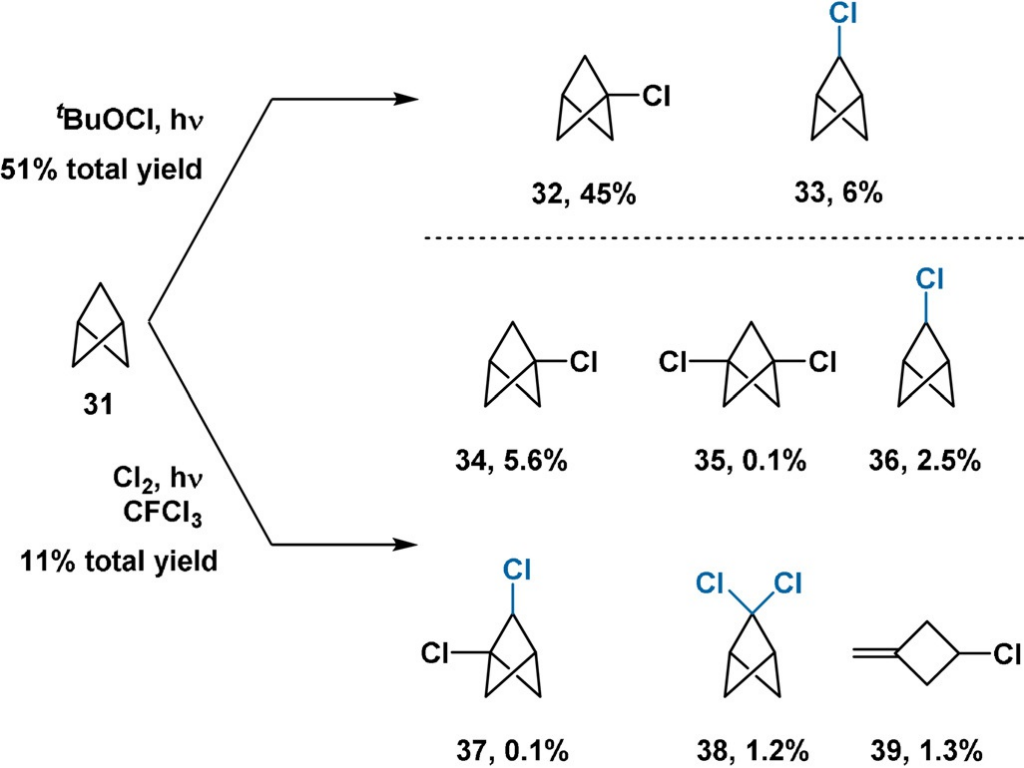
Direct chlorination of the parent BCP hydrocarbon.

The direct chlorination of BCP derivatives has been extensively investigated and represents the first reported *direct* functionalisation of the BCP core. Photochemical chlorination has been demonstrated using both ^*t*^BuOCl^[44]^ and elemental Cl_2_ ^[29]^ by Wiberg, with 22.5:1 and 7:1 selectivity for bridgehead:bridge substitution, respectively (Scheme 7). Notably, the preference for bridgehead substitution contrasts with the direct chlorination of other bicyclic hydrocarbons (e.g. norbornane) which typically give zero or little bridgehead substitution.^[29]^

Repeating the chlorination with Cl_2_ at higher chlorine concentration increased the total yield to 50 %, with a broadly similar product distribution except with zero production of **39**.^[29]^ This result was initially taken to affirm the kinetic stability of the BCP bridgehead radical: only when the supply of Cl_2_ is limited is the radical sufficiently long‐lived to rearrange to give the ring‐opened cyclobutane product. However, this was later disputed by Della and co‐workers,^[45]^ who argued that **39** instead arises from *heterolysis* of the bridgehead C−Cl bond of 1‐chlorobicyclo[1.1.1]pentane and rearrangement of the resulting cation.

**Scheme 8:**
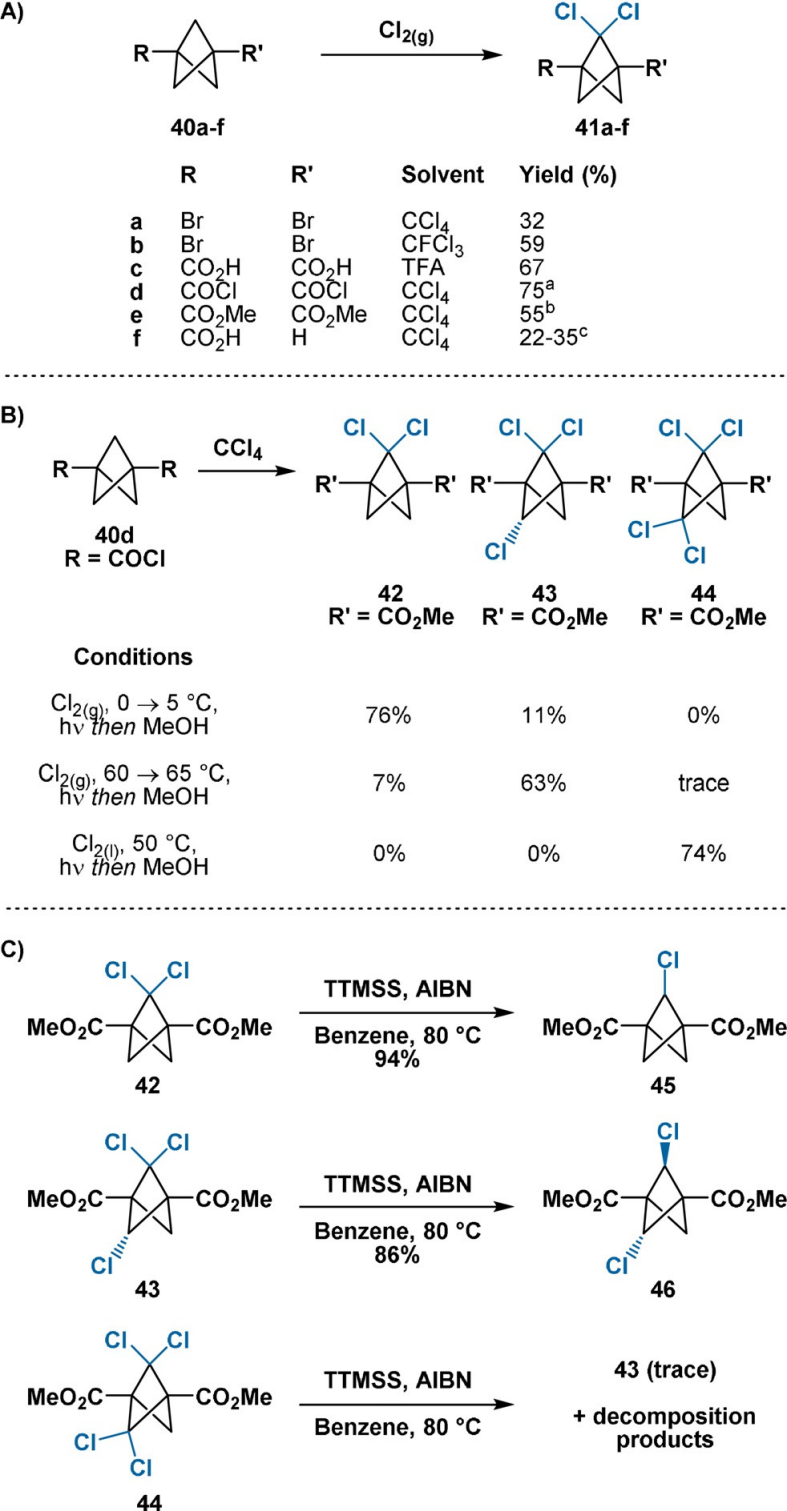
A, B): Direct chlorination of BCP derivatives. C): Protodechlorination of chlorinated BCP derivatives with TTMSS (tris(trimethylsilyl)silane). [a] Reaction mixture quenched with MeOH; yield refers to that of the obtained diester. [b] Hydrolytic workup then (COCl)_2_/MeOH esterification; yield refers to that of the obtained diester. [c] Isolated as the methyl ester through (COCl)_2_/MeOH esterification; yield refers to that of the obtained ester. AIBN=azobisisobutyronitrile; TFA=trifluoroacetic acid.

In 1989, Michl demonstrated the bridge 2,2‐dichlorination of 1,3‐disubstituted BCP derivatives with gaseous Cl_2_ (Scheme 8 A).^[30]^ Bicyclo[1.1.1]pentane‐1‐carboxylic acid, bearing no substituent at one bridgehead site, also underwent selective bridge dichlorination. As discussed in Section 2.2, this arises from the electron‐withdrawing carboxyl group decreasing the stabilisation conferred to the bridgehead radical through the 1,3‐transannular interaction. No significant quantities of monochlorinated products were observed in any case: this was rationalised through stabilisation of the transition state for the second HAT event by the installed chlorine atom. While these studies increase the synthetic utility of the previous work of Wiberg, they are still limited by the requirement for Cl_2_ gas and the use of CCl_4_, CFCl_3_ and TFA solvents.

More recently, the same group has also accessed tri‐ and tetrachlorinated BCP derivatives through direct chlorination (Scheme 8 B).^[43b]^ Elevated temperatures and gaseous Cl_2_ were required for effective trichlorination, while elevated temperatures and pressurised liquid Cl_2_ were required for tetrachlorination. The regio‐ and stereoselectivity of polychlorination was also rationalised. TTMSS/AIBN reduction of 2,2,5‐trichlorinated BCP **43** gave access to the 2,5‐ dichlorinated species, while attempted TTMSS/AIBN reduction of 2,2,5,5‐tetrachlorinated BCP **44** unexpectedly led only to decomposition products (Scheme 8 C).

**Scheme 9:**
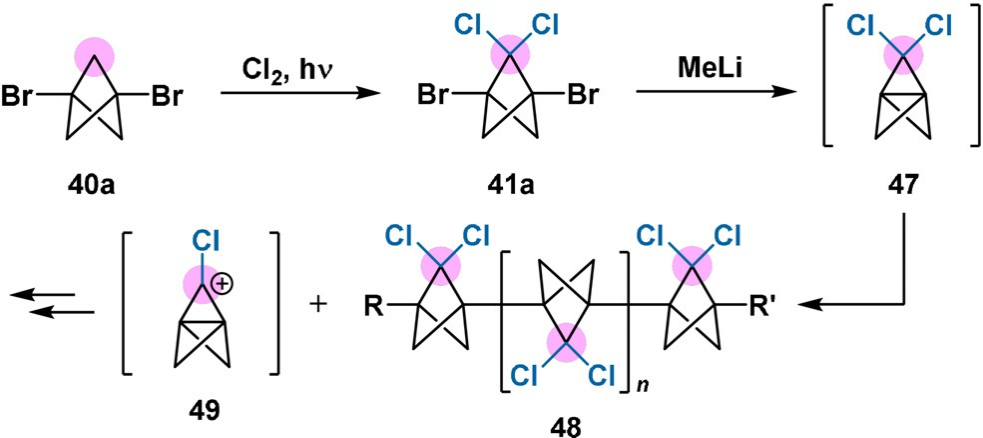
Preparation and decomposition of the metastable 2,2‐dichloro[1.1.1]propellane (**47**).

Finally, it should be noted that bridge‐chlorinated BCP derivatives likely cannot be accessed through functionalisation of 2,2‐dichloro[1.1.1]propellane (**47**). Compound **47**, prepared in 1992 by Michl and co‐workers,^[46]^ was found to be highly susceptible to oligomerisation and loss of a bridge chlorine atom, producing unstable cation **49** (Scheme 9). This loss of chlorine is consistent with that reported previously for a higher *gem*‐dichloropropellane.^[47]^ By analogy with computational studies on *gem*‐difluorinated BCP derivatives,[^17a^, ^48^] the instability of **47** could be rationalised through angular distortion of the propellane cage caused by geminal bridge substitution, which would favour an increased C‐C‐C valence angle at the bridge. This strain can be partially relieved either through oligomerisation to **48**, or through loss of chloride and subsequent cage rearrangement. It is therefore postulated that other propellanes with bridge substituents that are competent leaving groups will also be unstable.^[31a]^

### 4.2 Bridge‐Fluorinated Derivatives

Bridge‐fluorinated BCP derivatives have garnered significant interest in the past two years. Unsurprisingly, direct fluorination of 1,3‐disubstituted BCPs with limited gaseous F_2_ is extremely unselective, producing 14 out of the 15 possible bridge‐fluorinated derivatives.^[48]^ Exhaustive fluorination gives access to penta‐ and hexafluorinated derivatives in synthetically useful yields.^[25b]^ Recent attempts^[31a]^ at controlled fluorination on the BCP core through the electrochemical method of Baran,^[49]^ and the amine‐directed, Pd‐catalysed approaches of Xu^[50]^ and Hrdina,^[51]^ have all proven unsuccessful. Only via difluorocarbene insertion have bridge‐fluorinated BCP derivatives been prepared in a controlled fashion.

**Scheme 10:**
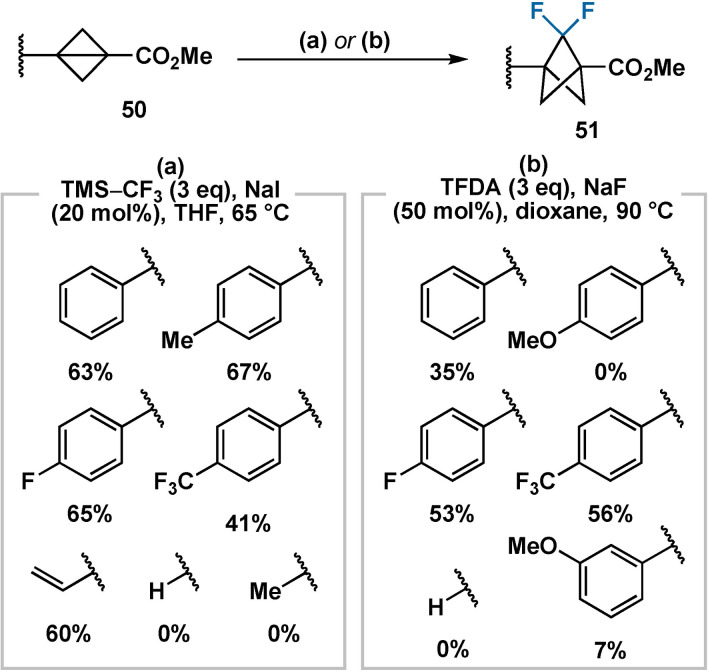
Preparation of 2,2‐difluorinated BCP derivatives through insertion of difluorocarbene into bicyclo[1.1.0]butanes.

The concurrent works of Mykhailiuk^[31a]^ and Ma^[52]^ disclosed the first examples of this chemistry in 2019, using the Ruppert–Prakash reagent (TMS‐CF_3_) and the Dolbier reagent (TFDA, FSO_2_CF_2_CO_2_TMS) as difluorocarbene precursors, respectively (Scheme 10). Anderson has also recently demonstrated difluorocarbene insertion using a zwitterionic phosphonium carboxylate precursor.^[53]^ Preliminary results towards the generation of 2‐chloro‐2‐fluorobicyclo[1.1.1]pentane derivatives, using CHFCl_2_ as a chlorofluorocarbene source, were also presented by Mykhailiuk. Across both reports, the reaction scope was limited to aryl‐ or vinyl‐substituted bicyclo[1.1.0]butanes, a limitation apparently not shared by the related dichlorocarbene insertion reaction.^[54]^ However, between the two reports, fluorinated BCP derivatives bearing both electron‐neutral and electron‐poor aromatic substituents are accessible (Scheme 10). Bicyclo[1.1.0]butanes bearing electron‐rich aromatic substituents are not viable substrates on account of their instability with respect to ring opening, even at room temperature.

The stability of the difluorinated BCP motif to acidic, basic, reducing and forcing palladium cross‐coupling conditions was also demonstrated between the two reports. Although vinyl or aryl substituents were essential for the difluorocyclopropanation, both can subsequently be oxidised to carboxylic acids[^31a^, ^54^] and further derivatised. This was later exploited in a further publication by Ma in 2020,^[55]^ demonstrating various transformations of *gem*‐difluorinated BCPs at the bridgehead positions.

A further general limitation of dihalocarbene insertion reactions arises from the nature of the synthesis of the starting materials. An electron‐withdrawing group (or a halogen) is required in intermediates **3**, so as to facilitate deprotonation or metal–halogen exchange to enable the required intramolecular cyclisation reaction. Access to the cyclobutanol intermediates (**2**) is also nontrivial, as the addition of sterically hindered, electron‐poor and heteroaryl Grignard reagents to cyclobutanones is challenging.^[52]^ Ma and co‐workers have, however, illustrated the construction of five‐membered heteroaryl rings appended to the difluoro‐BCP core at a late stage using classical carbonyl‐centred ring syntheses.^[55]^

## 5 Carbon Substituents

BCP derivatives bearing bridge carbon substituents have been accessed in four fundamental ways: through chlorocarbonylation of a BCP precursor; through NY cyclisation of cyclobutyl aryl ketones; from substituted [1.1.1]propellanes; and from intramolecular nucleophilic displacement on a substituted cyclobutane. The synthesis of tricyclo[2.1.0.0^2,5^]pentanes (where two bridge positions on the BCP core are directly bonded), and miscellaneous synthetic routes that afford bridge carbon‐substituted BCP derivatives as minor products, are beyond the scope of this review. Both topics are addressed by Michl.^[17a]^

The installation of carbon substituents on the BCP core through intermolecular carbene insertion into a bicyclo[1.1.0]butane (Scheme 1) has not yet been demonstrated, although there are a small number of reports of a related intramolecular reaction providing [1.1.1]propellanes (see Section 5.3). Attempts at intermolecular delivery of a CH_2_ fragment via photolysis with diazomethane^[56]^ and under Simmons–Smith conditions have been unsuccessful.[^13d^, ^57^] Attempted reaction of bicyclo[1.1.0]butanes with bis(carbomethoxy)carbene was also unsuccessful.^[58]^ Experimental and computational studies of the mechanism of carbene insertion into bicyclo[1.1.0]butanes have been previously discussed.[^17a^, ^58^]

### 5.1 Carbon Substitution through Direct Chlorocarbonylation

**Scheme 11:**
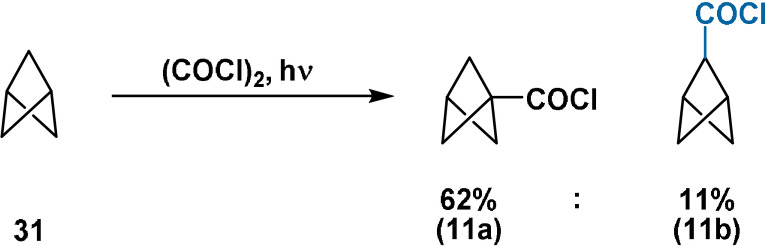
Preparation of acyl chlorides **11 a**,**b** through photochemical chlorocarbonylation of the parent BCP hydrocarbon.

Acyl chloride **11 b** represents the first example of a BCP derivative bearing a bridge carbon substituent. The product distribution of chlorocarbonylation of **31** with oxalyl chloride corresponds to a selectivity of 17:1 for bridgehead:bridge substitution. As described previously, manipulation of mixed acyl chlorides **11** also gave first access to bicyclo[1.1.1]pentan‐2‐ol (**14 b**) (Scheme 11).

### 5.2 Carbon Substitution through Norrish–Yang (NY) Cyclisation

**Scheme 12:**
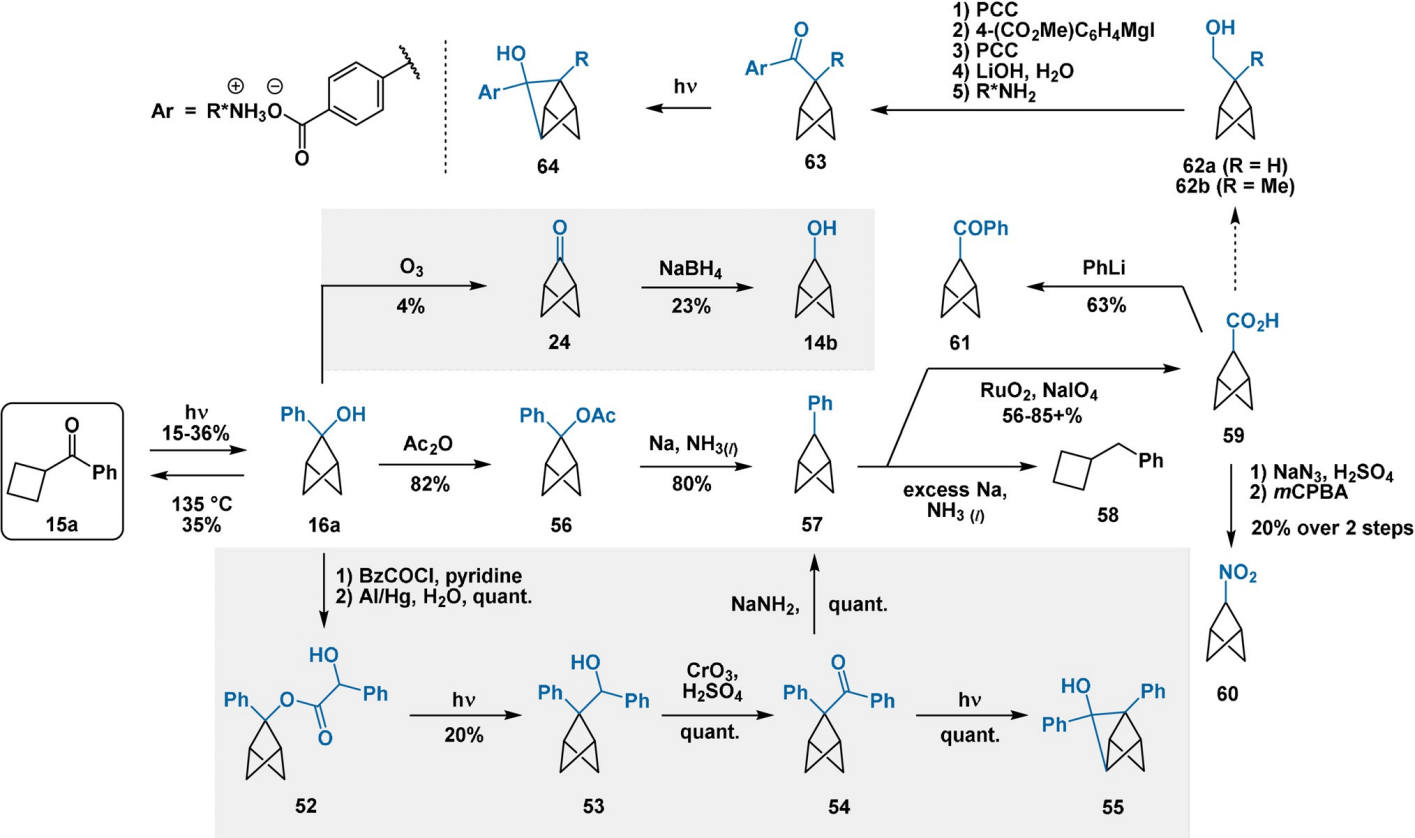
NY cyclisation of cyclobutyl phenyl ketone and manipulation of resulting alcohol **16 a**. The synthesis of compounds **14 b/24** and **60** (described in Sections 3.1 and 6, respectively) are also included for clarity. “quant”=quantitative; R*=enantiopure chiral fragment.

The synthetic utility of the NY cyclisation of cyclobutyl phenyl ketone (Section 3.1) was explored by Alexander in the 1970s^[59]^ and later in 1993^[35b]^ by Wiberg (Scheme 12). In two steps from alcohol **16 a**, Alexander prepared mandelate ester **52** which underwent photochemical extrusion of CO_2_ to afford **53**. Jones oxidation then provided ketone **54**. Under irradiation,^[59a]^ a second NY cyclisation afforded tricyclo[2.2.0.0^2,5^]hexanol **55**. Alternatively, treatment with NaNH_2_ ^[59b]^ effected formal Haller–Bauer cleavage to afford 2‐phenylbicyclo[1.1.1]pentane (**57**). Presumably the generation of an intermediate *benzylic* anion promotes formation of **57** rather than the bicycloalkyl carboxamide, the typical product of the Haller–Bauer reaction. Compound **57** was more conveniently accessed by Wiberg^[35b]^ through acylation of **16 a** and subsequent reduction with metallic sodium.

Direct deoxygenation of **16 a** with Li/NH_3_ was unsuccessful due to rapid cycloreversion to cyclobutyl phenyl ketone (**15 a**) under basic conditions (see Section 3.1). Reductive deoxygenation of **16 a** using Et_3_SiH/BF_3_⋅Et_2_O and halogenative deoxygenation under Appel conditions were also unsuccessful.^[35b]^ Both resulted in fragmentation of the BCP core, further corroborating the instability of the putative bridge carbocation. Both Alexander and Wiberg demonstrated oxidation of the aromatic ring of **57** to the carboxylic acid, which provided access to 2‐benzoylbicyclo[1.1.1]pentane (**61**) and 2‐nitrobicyclo[1.1.1]pentane (**60**) via bicyclo[1.1.1]pentyl‐2‐amine.

Across two studies in 2005^[60]^ and 2012,^[61]^ Xia, Yang et al. reported findings from a study of enantioselective photochemical solid‐state NY cyclisations of ketones **63**. Asymmetry originates from the homochiral environment of the crystalline lattice of the solid salts. Ketones **63** were prepared from alcohols **62**, presumably originating from carboxylic acid **59** or a derived ester. Ultraviolet irradiation of the solid ketones gave the corresponding tricyclic alcohols **64** with modest enantioselectivity (maximum 90 %^[60]^ and 60 %^[61]^ *ee*). Extended reaction times and/or higher reaction temperatures were necessary to achieve satisfactory conversion in the solid state; however, increased conversion was generally to the detriment of asymmetric induction.

### 5.3 Carbon Substitution through the Use of Substituted [1.1.1]Propellanes

[1.1.1]Propellanes are attractive intermediates for the synthesis of BCP derivatives, as their unique electronic profile^[62]^ allows opening of the central bond with a broad variety of carbon‐ and heteroatom‐centered radicals and nucleophiles (treatment with electrophiles^[62]^ or transition metal catalysts^[63]^ affords exclusively ring‐opened products). As such, [1.1.1]propellanes could represent key divergent intermediates for the library synthesis of BCP derivatives in drug discovery. A variety of bridge‐substituted [1.1.1]propellanes have been prepared, including 2,2′‐bonded dimers and those featuring spirocyclic attachment of another ring, either through a polar cyclisation strategy^[64]^ or through intramolecular carbene insertion.^[65]^ However, many of these substituents are simple hydrocarbons or contain few functional handles for further derivatisation.

Furthermore, relatively few substituted propellanes have then been opened to afford monomeric bicyclo[1.1.1]pentane derivatives.[^64a^, ^64d^, ^64e^, ^64f^, ^64g^, ^64h^, ^65a^, ^66^] Some have been polymerised to afford [*n*]staffane chains; this has been recently reviewed by Bräse.^[67]^ Co‐polymerisation of substituted propellanes with acrylates has also been studied, as well as the preparation of bridge‐substituted‐BCP‐containing polyamide polymers.^[67]^

Simple examples of the functionalisation of bridge‐substituted propellanes were provided by Szeimies,[^64a^, ^65a^] with more recent advances from the groups of Kaszynski,^[64f]^ Mazal,[^64g^, ^64h^] Baran^[31b]^ and Ma.^[68]^

**Scheme 13:**
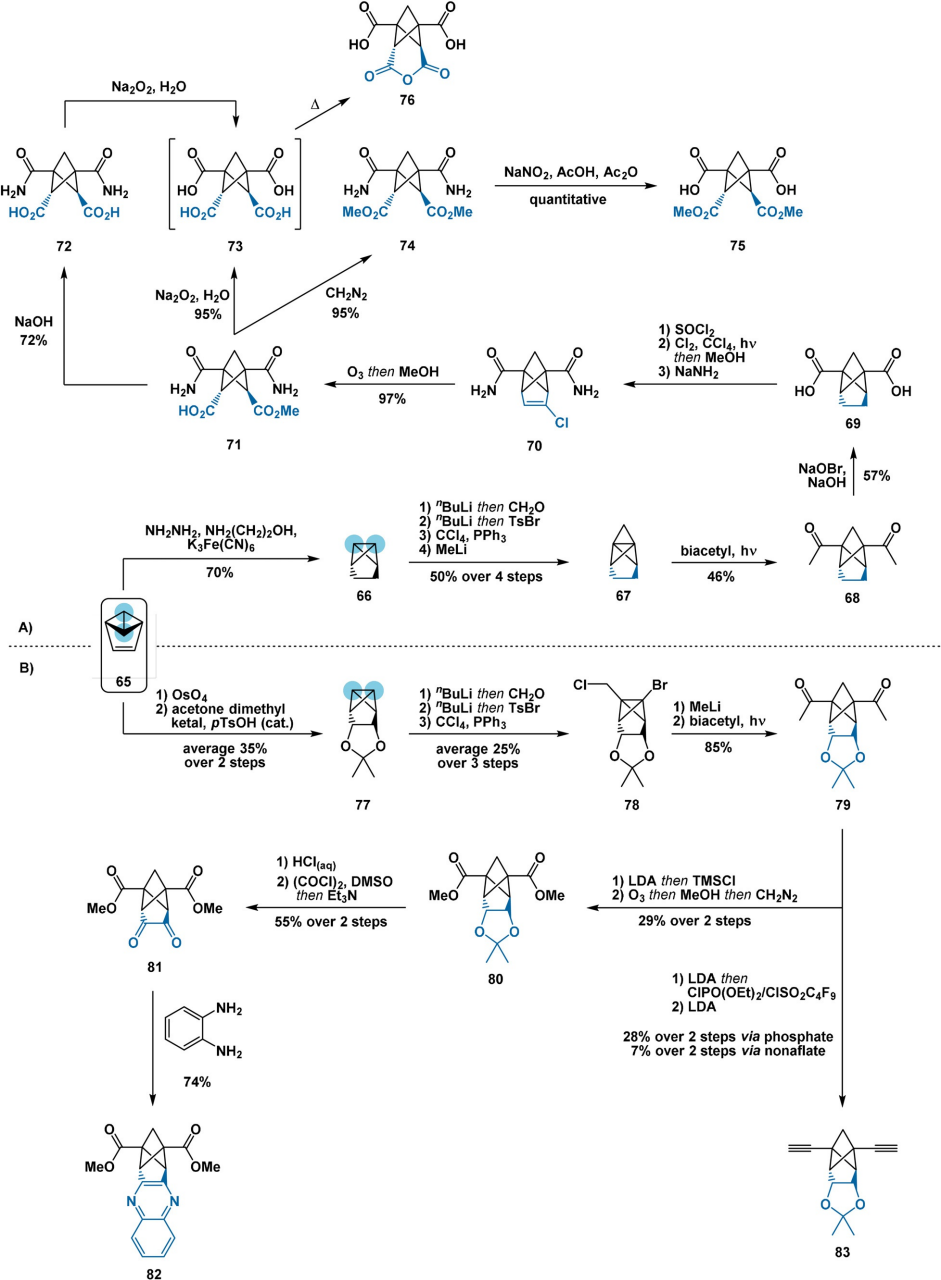
Synthesis of bridge‐substituted BCP derivatives via substituted propellanes prepared from benzvalene. LDA=lithium diisopropylamide; TMS=trimethylsilyl; *p*TsOH=*p*‐toluenesulfonic acid; nonaflate=nonafluorobutanesulfonate.

Kaszynski^[64f]^ and Mazal[^64g^, ^64h^] demonstrated the preparation of 1,2,3,4‐tetrasubstituted BCP derivatives via functionalisation of substituted [1.1.1]propellanes accessed from benzvalene (**65**). In one approach (Scheme 13 A),^[64g]^ diimide reduction of benzvalene afforded alkane **66**. The acidity of the bicyclo[1.1.0]butane scaffold then enabled construction of the propellane motif in four steps according to the original method of Szeimies.^[64a]^ Photochemical radical opening with biacetyl furnished intermediate **68**, whose acyl groups were oxidised via haloform reaction to provide diacid **69**. Subsequent treatment with liquid Cl_2_ resulted in exclusive halogenation of the two‐carbon bridge over the BCP core, consistent with the BDE trends described previously (Section 2.2). Treatment of the obtained mixed chlorides with sodium amide furnished olefin **70**, which underwent ozonolysis in high yield to afford *cis*‐*endo*‐tetrasubstituted BCP derivative **71**. Compound **71** then served as a common precursor for the synthesis of four other tetrasubstituted analogues (**72**–**75**) through conventional carbonyl manipulations. Acid **73** spontaneously formed anhydride **76** during isolation, which went to completion on heating above 80 °C for several hours.

In the second approach (Scheme 13 B),[^64f^, ^64h^] osmium‐mediated dihydroxylation of **65** followed by ketalisation delivered dioxolane **77**. Construction of the propellane motif, then photochemical radical opening with biacetyl furnished bridged BCP **79**. After oxidation to **80**,^[64h]^ the dioxolane moiety was deprotected prior to Swern oxidation to furnish unstable 1,2‐diketone **81**. Condensation of **81** with *o*‐phenylenediamine afforded stable BCP‐fused quinoxaline **82**. Alternatively, the ketone moieties of **79** were transformed into vinyl phosphates or nonaflates, whereupon elimination with LDA furnished bis‐alkyne **83**.

**Scheme 14:**
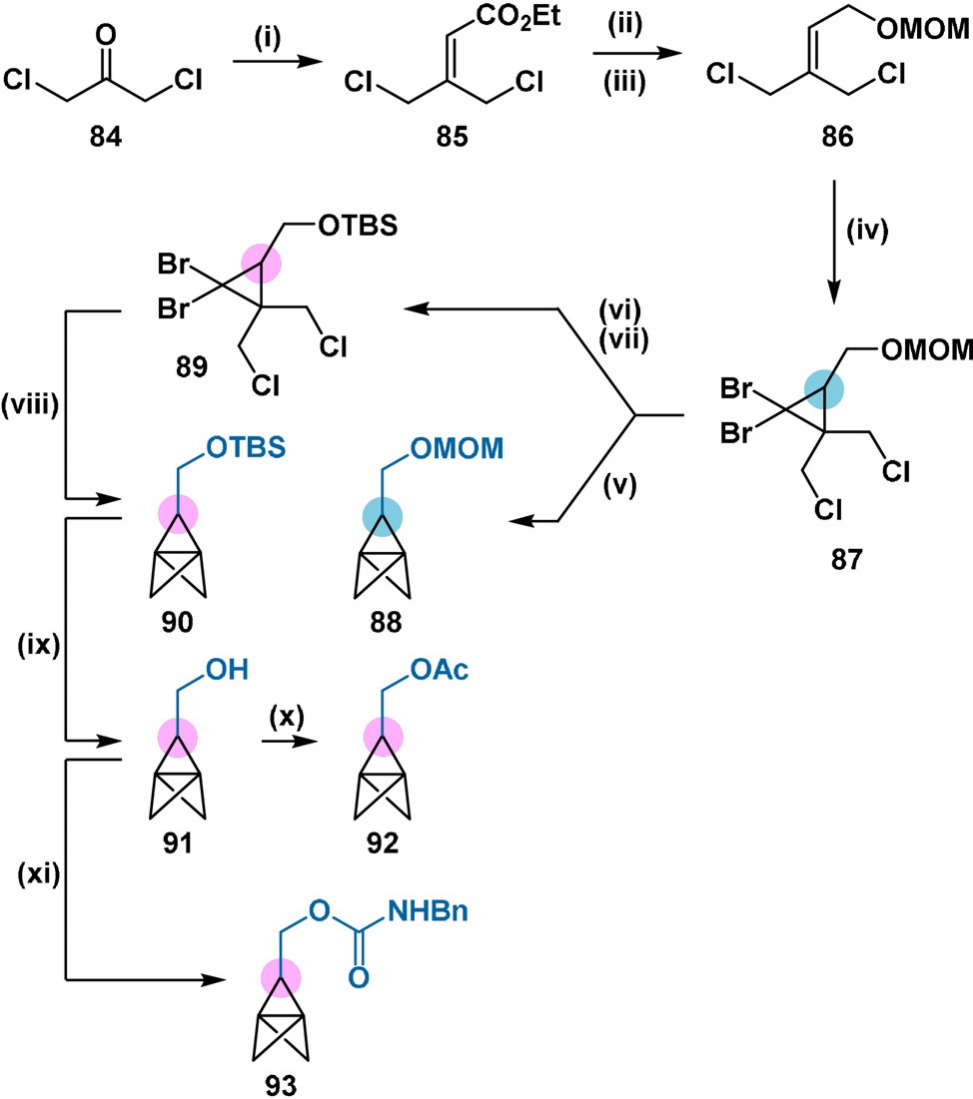
Synthesis of propellanes **88** and **90**–**93**. Reagents and conditions: i) NaH, triethyl phosphonoacetate, 83 %; ii) LiAlH_4_, AlCl_3_, 90 %; iii) dimethoxymethane, P_2_O_5_, 90 % iv) CHBr_3_, NaOH, PhMe_3_N^+^Cl^−^, 30 %; v) MeLi, 57 %; vi) HCl, MeOH, 90 %; vii) TBSCl, imidazole, 95 %; viii) MeLi, 55 %; ix) TBAF; x) Ac_2_O, Et_3_N, DMAP; xi) BnNCO, Et_3_N.

In 1993 and 1995 reports, Schlüter and co‐workers reported the synthesis of five propellanes containing a protected and free hydroxymethyl functionality (Scheme 14).[^64d^, ^64e^] These were accessed through a modified Szeimies procedure employing functionalised olefin **86**. Aside from the synthesis of [*n*]staffane polymers, the synthetic chemistry of these propellanes would surprisingly not be explored until late 2020 in the works of Ma^[68]^ and Baran.^[31b]^

**Scheme 15:**
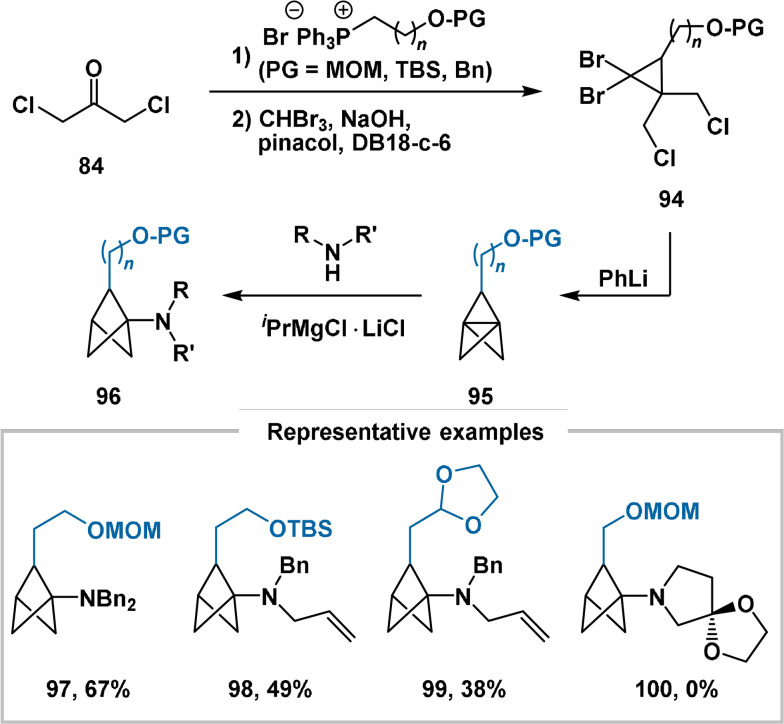
Preparation of C2‐functionalised bicyclo[1.1.1]pent‐1‐ylamines. DB18‐c‐6=dibenzo‐18‐crown‐6.

The work of Ma delineated the synthesis of analogues of propellane **88** through a similar method. The generated propellanes (**95**) were then subjected to the strain‐release amination conditions of Baran,^[69]^ furnishing 1‐amino‐2‐functionalised BCP products (**96**) (Scheme 15). MOM, TBS and benzyl *O*‐protecting groups were all compatible with the synthetic sequence. A dioxolanylmethyl substituent at C2 was also tolerated. A small range of secondary amines were proficient substrates, including allylic/benzylic amines and piperidine derivatives; however, sterically hindered amines, cyclopropylamines and pyrrolidine derivatives were unsuccessful. After methoxymethyl cleavage, the alcohol functionality of bicyclopentane **97** was derivatised to the corresponding fluoride, azide, thioacetate and *N*‐(4‐fluorophenyl)carbamate under standard conditions.

These studies represent the first peer‐reviewed investigation into a convenient and general strategy for the synthesis of bridge‐substituted BCP derivatives. However, the chemistry is currently limited by its poor performance with more complex amines and the difficulty of achieving simultaneous 1,3‐disubstitution across the propellane: preliminary attempts at aminoalkylation under the conditions of Gleason,^[70]^ and aminoalkenylation under the Zweifel conditions of Aggarwal,^[71]^ were unsuccessful and extremely low‐yielding, respectively. For the bioisosteric replacement of an *ortho‐* or *meta*‐substituted benzenoid fragment this may not be necessary, but having equal access to both 1,2‐disubstituted and 1,2,3‐trisubstituted BCP derivatives would greatly increase the synthetic utility of the chemistry and broaden the chemical space it can access. Restricting studies to the synthesis of 1,2‐disubstituted BCP derivatives can arguably never fully capitalise on the three‐dimensionality of the BCP core.

**Scheme 16:**
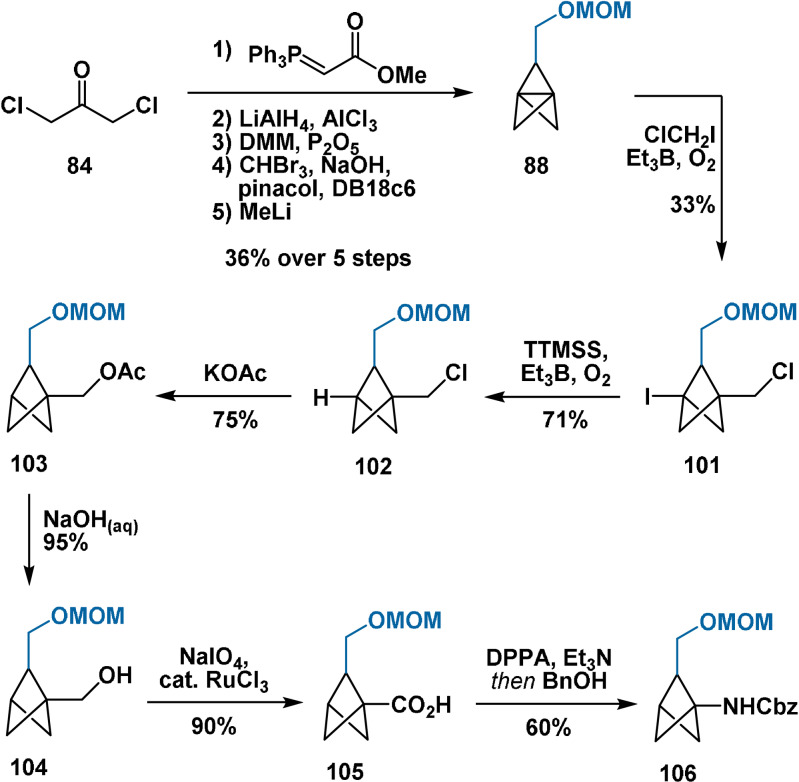
Preparation of compounds **101**–**106**. DMM=dimethoxymethane; DPPA=diphenylphosphoryl azide.

In the report of Baran, propellane **88** was prepared analogously to the Schlüter approach; treatment with chloroiodomethane under the conditions of Anderson^[72]^ then delivered compound **101** in 33 % yield (Scheme 16). Radical de‐iodination provided **102**, whose chloromethyl handle was then derivatised to the corresponding acetate (**103**), alcohol (**104**), carboxylic acid (**105**) and Cbz‐protected amine (**106**).

**Figure 1:**
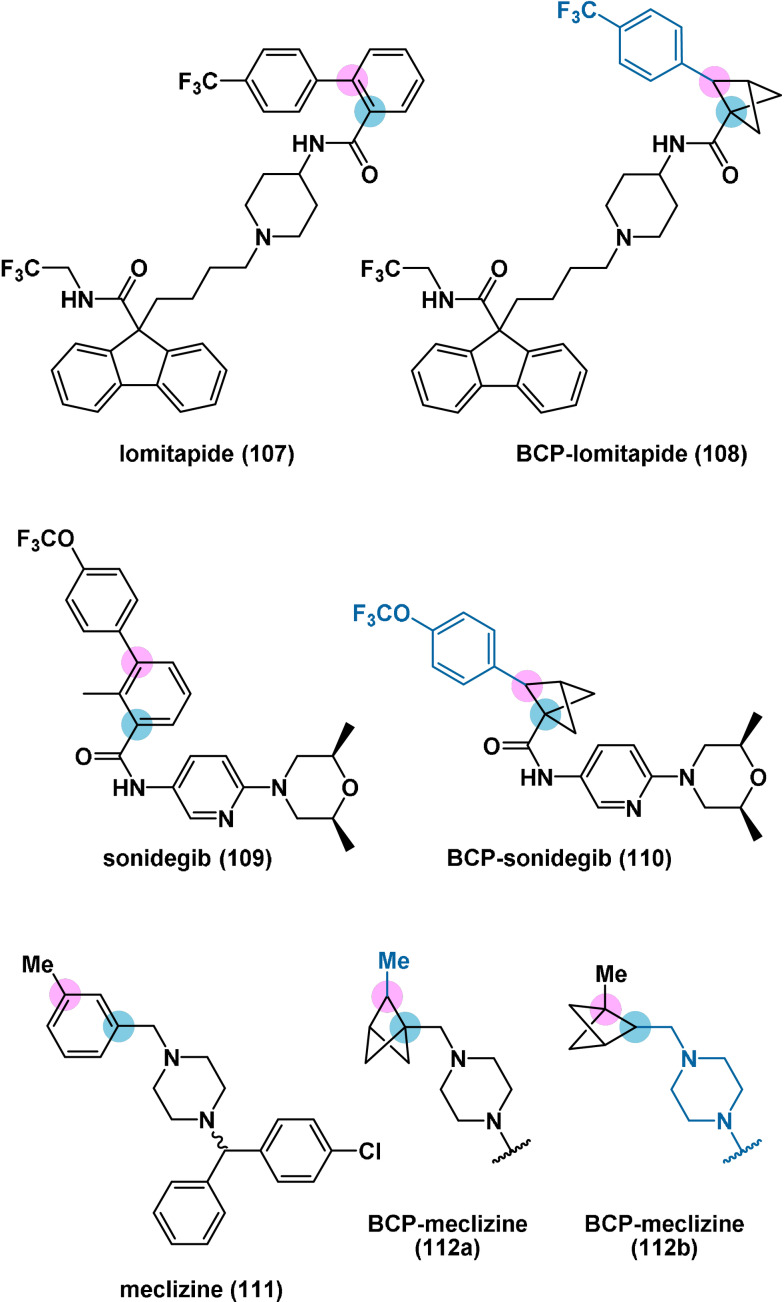
Structures of lomitapide (**107**), sonidegib (**109**) and meclizine (**111**) and their BCP‐containing analogues (**108**, **110**, and **112 a/b**, respectively).

Compounds **103** and **104** were further used to prepare analogues of six drugs containing *ortho*‐ or *meta*‐substituted benzene rings, including MTP inhibitor lomitapide (**107**), Hh signalling inhibitor sonidegib (**109**) and histamine H_1_ antagonist meclizine (**111**) (Figure 1). For the latter and three other examples, both constitutional isomers about the BCP framework were prepared, differing in which substituents of the benzene ring occupy the bridge and bridgehead positions.

**Scheme 17:**
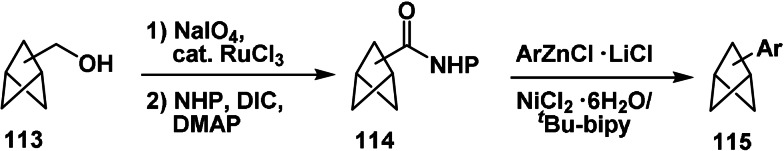
General approach to arylation of the BCP core through decarboxylative Negishi arylation. DIC=diisopropylcarbodiimide; DMAP=4‐(dimethylamino)pyridine.

For the direct appendage of aromatic rings to the BCP core, conversion of BCP carboxylic acids to *N*‐hydroxyphthalimide (NHP) esters permitted application of the group's thermal decarboxylative Negishi arylation chemistry (generalised in Scheme 17).

In summary, the work of Baran broadens the range of synthetic transformations known for decoration of the BCP core, and provides the first examples of bridge‐substituted BCP analogues of drug compounds. Future work will help elucidate the *ortho* vs. *meta* character of 1,2‐disubstituted BCPs in this context. The work confirms the viability of thermal decarboxylation of redox‐active esters on the bridge positions of BCPs (previously demonstrated only on the bridgehead positions), but the full synthetic potential of the generated radical is yet to be explored. As discussed, future work focusing on 1,2,3‐trisubstitution of the BCP system will further increase the value of the developed methods within drug discovery.

### 5.4 Carbon Substitution through Intramolecular Nucleophilic Displacement

**Scheme 18:**
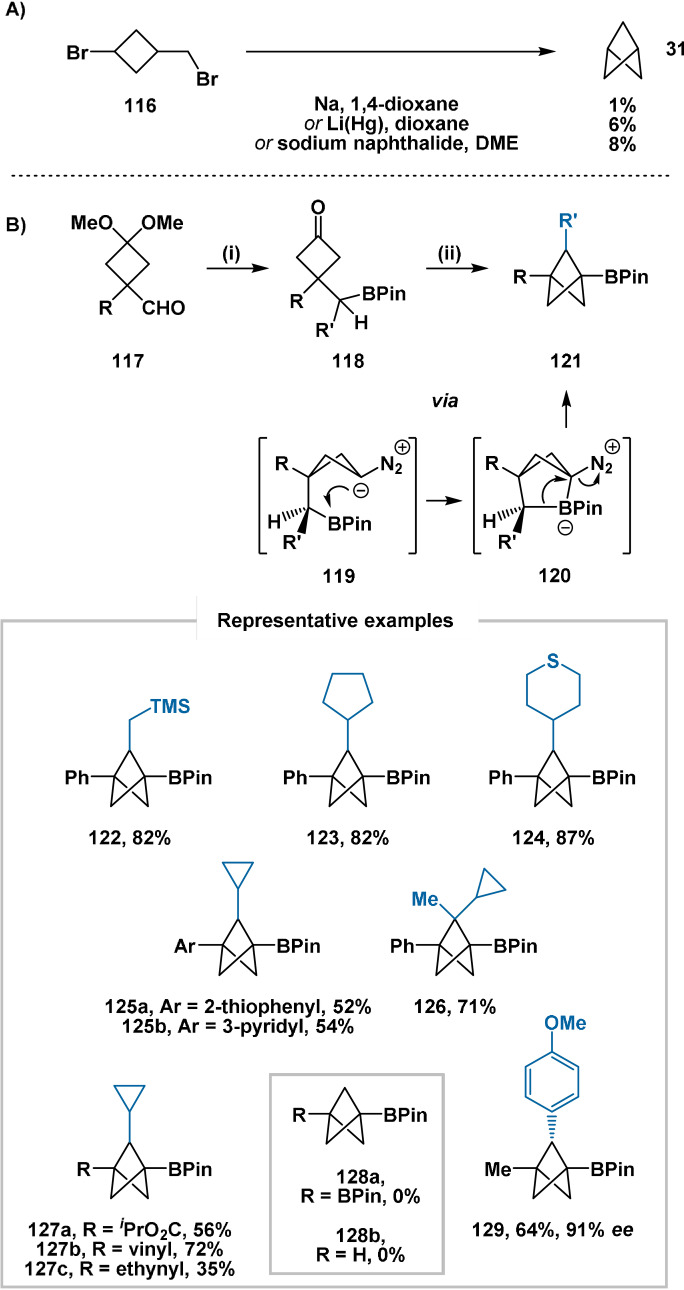
A): Original preparation of the parent BCP hydrocarbon through intramolecular Wurtz coupling. DME=1,2‐dimethoxyethane. B): Synthesis of BCP derivatives **121**. Reagents: i) MesSO_2_NHNH_2_, R′B(OH)_2_, pinacol *then* aqueous H_2_SO_4_; ii) MesSO_2_NHNH_2_, Cs_2_CO_3_, 100 °C.

In early 2021, Qin presented an elegant contemporary adaptation (Scheme 18 B)^[73]^ of the original intramolecular displacement methodology employed in the first synthesis of bicyclo[1.1.1]pentane (Scheme 18 A).[^44^, ^74^] Cyclobutanones **118** were accessed through the group's boron‐preserving cross‐coupling of aldehydes **117** followed by ketal hydrolysis. Conversion of **118** to mesitylsulfonyl hydrazones, followed by addition of Cs_2_CO_3_, furnished intermediate diazo compounds **119**. Trapping of the pendant boron atom delivered high‐energy zwitterionic intermediates **120**, which rearranged to afford 2‐substituted bicyclo[1.1.1]pentanes **121** via 1,2‐metalate rearrangement with extrusion of dinitrogen. A key benefit of the developed process lies in its robustness: in contrast to reactions employing propellanes, this reaction can be performed in air with only a small decrease in yield. Additionally, as stoichiometric water is generated during sulfonylhydrazone formation (Scheme 18 B, step ii)), the reaction is entirely tolerant of trace water in reagents and solvents.

A range of secondary BPin esters were viable substrates, providing bridge alkyl‐ (**122**) and cycloalkyl‐ (**123**–**125**) substituted BCP derivatives. A primary BPin ester could also be cyclised to afford the bridge‐unsubstituted product. Tertiary BPin esters also successfully underwent cyclisation, providing geminal bridge‐disubstituted products (e.g. **126**). Heteroaryl (**125**), isopropyl ester (**127 a**), vinyl (**127 b**), ethynyl (**127 c**), methyl, amide and carbamate groups were tolerated as substituents on the cyclobutane ring (R). A positive correlation was identified between the steric demand of the “R” substituent and the reaction yield: this is a consequence of increased preference for the BPin‐containing side chain to adopt the pseudo‐axial position required for cyclisation. This requirement means that cyclobutanones with small “R” substituents are not viable substrates: compounds **128 a**,**b**, with R=BPin (A‐value=0.42 kcal mol^−1^)^[75]^ and R=H, respectively, were not accessible using this methodology. The introduction of a bridge methyl substituent was also demonstrated through α‐substitution on the cyclobutanone precursor, rather than substitution adjacent to the boron centre. Finally, asymmetric borylation under the conditions of Aggarwal provided an enantioenriched secondary BPin ester cyclisation precursor, which was cyclised with only 3 % erosion of enantiopurity to afford BCP derivative **129**. To our knowledge, this represents the first example of asymmetric functionalisation of the bridge positions of BCP derivatives.

The BPin group of compound **123** was derivatised to hydroxyl, vinyl and heteroaryl functionalities under standard conditions. Matteson reaction^[76]^ provided the homologated primary BPin ester, while conversion to the trifluoroborate salt further enabled proto‐deboronation and Suzuki cross‐coupling reactions. The trifluoroborate salt could also be employed in another round of boron‐preserving cross‐coupling chemistry with a ketone, providing another means of formal homologation of the C−B bond.

In summary, the work of Qin provides a conceptually distinct method for the synthesis of bridge‐substituted BCP derivatives. The developed methodology is operationally simple and can deliver nonsymmetrical 1,3‐di‐, 1,2,3‐tri‐, and 1,2,2,3‐tetrasubstituted BCP products containing versatile functional handles for downstream derivatisation. However, a limitation is arguably its reduced divergency compared to the methods of Baran and Ma, wherein a [1.1.1]propellane provides an early common intermediate to potentially hundreds of unique derivatives. With the present method, bespoke cyclisation precursors must be synthesised (asymmetrically, if required) for each fundamental series of compounds, limiting its applicability to late‐stage functionalisation. Only the BCP products post‐cyclisation represent points of divergency.

## 6 Nitrogen Substituents

**Scheme 19:**
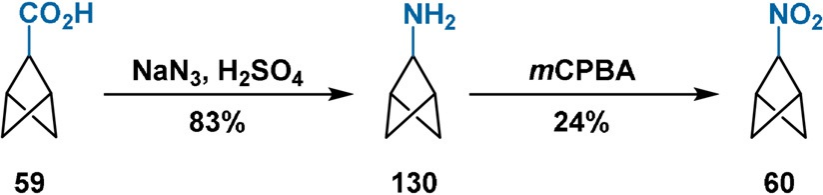
Schmidt rearrangement of acid **59** to amine **130** and subsequent oxidation to 2‐nitrobicyclo[1.1.1]pentane (**60**).

Surprisingly, none of the works of Baran, Ma or Qin considers the synthesis of BCP derivatives containing bridge nitrogen substituents. This is in contrast to the considerable progress made towards installation of nitrogen atoms at the bridgehead positions,[^17b^, ^69^, ^70^, ^77^] for example, for bioisosteric replacement of anilines, common structural alerts and toxicophores in drug design. To date, nitrogen has only been installed on the bridge positions of BCP derivatives through Schmidt rearrangement of carboxylic acid **59** (Schemes 12 and 19) by Wiberg.^[35b]^ Alexander previously reported the same transformation in 1978;^[59b]^ however, this claim was not substantiated by characterisation data for the amine product. As expected, the electron‐withdrawing BCP core decreases the basicity of **130** compared to other cycloalkylamines.

Peroxyacid oxidation of **130** then furnished the corresponding nitro compound (**60**) in 24 % yield.^[35b]^ Like nitrocyclopropane and in contrast to nitrocyclopentane and nitrocyclohexane, **60** was found to be insoluble in aqueous NaOH. This is attributed to its increased p*K*_a_ compared to other nitrocycloalkanes, a consequence of the reluctance of the bridge carbon atom to engage in π‐bonding with associated increase in strain.

## 7 Boron Substituents

**Scheme 20:**
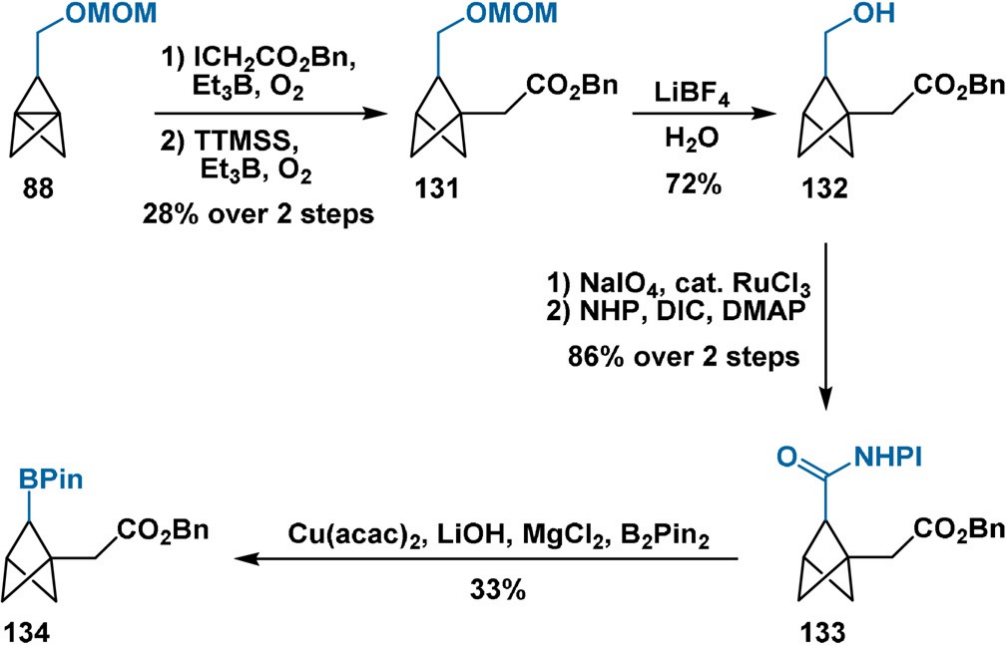
Synthesis of borylated BCP derivative **134** from [1.1.1]propellane **83**.

As part of their synthetic campaign, Baran and co‐workers aimed to establish a route to a boron‐containing BCP as a high‐value building block.^[31b]^ After propellane **88** was opened with benzyl iodoacetate, borylated BCP **134** was accessible via the group's copper‐catalysed decarboxylative borylation methodology in five linear steps (Scheme 20). No transformations of **134** were then explored; however, the sequence further validates decarboxylative radical transformations as a general manifold for the bridge functionalisation of BCP derivatives.

**Scheme 21:**
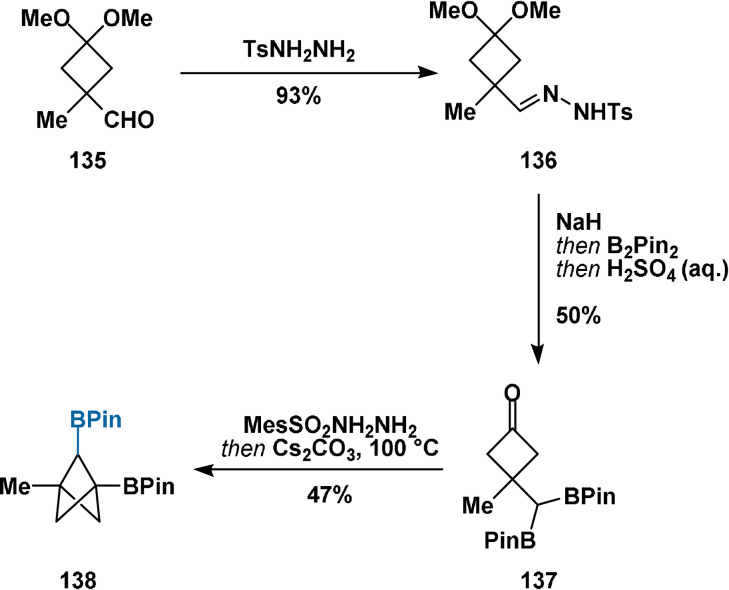
Synthesis of diborylated BCP **138** through the method of Qin and co‐workers.^[73]^

The methodology of Qin^[73]^ also provided BCP derivative **133** containing a bridge BPin substituent (Scheme 21). Likewise, no transformations of the bridge functionality were then explored.

## 8 Summary and Outlook

The works of Baran, Ma and Qin highlight two general synthetic strategies with potential to address the long‐standing question of the preparation of bridge‐functionalised BCP derivatives. The use of a substituted [1.1.1]propellane intermediate could facilitate divergent synthesis through the exploitation of its broad and ambiphilic reactivity profile. The application of decarboxylative radical transformations, dozens of which have now been reported,^[78]^ adds a further dimension of divergency. This is in stark contrast to the prior art in this field, which has been sporadic and largely reliant on inconvenient and low‐yielding synthetic methods.

With decarboxylative radical transformations on the BCP core successfully demonstrated, in the near future we are likely to witness the pursuit of a greater range of related transformations. The application of enabling technologies such as photoredox catalysis and synthetic electrochemistry will likely be central to this development. As such transformations often proceed under very mild conditions,^[79]^ the late‐stage bridge functionalisation of BCP derivatives may become commonplace. Judicious selection of different radical precursor groups, or the identification of synthetic sequences that allow the installation of a single radical precursor group at different stages, may also prove to be an effective strategy for the functionalisation of multiple bridge positions. In addition, the deployment of dual catalysis platforms such as metallaphotoredox catalysis, organophotoredox catalysis and mediated electrochemistry, could provide access to bridge substituted BCPs in an asymmetric fashion. This would provide a complementary approach to that illustrated by Qin, and would not require the synthesis of enantioenriched precursors.

Efficient asymmetric access to densely functionalised bicyclo[1.1.1]pentanes is likely to be transformational within drug discovery. The inclusion of such moieties into lead compounds increases their 3D character and stereochemical complexity without significantly increasing molecular weight, allowing a deeper exploration of chemical space and the design of molecules which can better complement the spatial subtleties of target proteins.[^12b^, ^80^] Increased potency and selectivity of lead compounds may reduce off‐target effects: drug promiscuity commonly results in adverse toxicity, a major cause of drug attrition in the clinic.^[12b]^ “Escape from flatland” promises to be a powerful solution to this widespread and intractable problem: we are apparently on the verge of a new era in the synthetic chemistry of the bicyclo[1.1.1]pentane core.

## Conflict of interest

The authors declare no conflict of interest.

## Biographical Information

*Joseph Anderson completed his MChem in 2020 (University of Oxford, Prof. Timothy Donohoe) working on cobalt‐catalysed transformations of boronic acids. He subsequently joined the GSK/University of Strathclyde Collaborative Industrial PhD Programme, where he is currently working under the supervision of Dr. Darren Poole, Dr. Nicholas Measom and Prof. John Murphy on bicyclo[1.1.1]pentane methodology*.

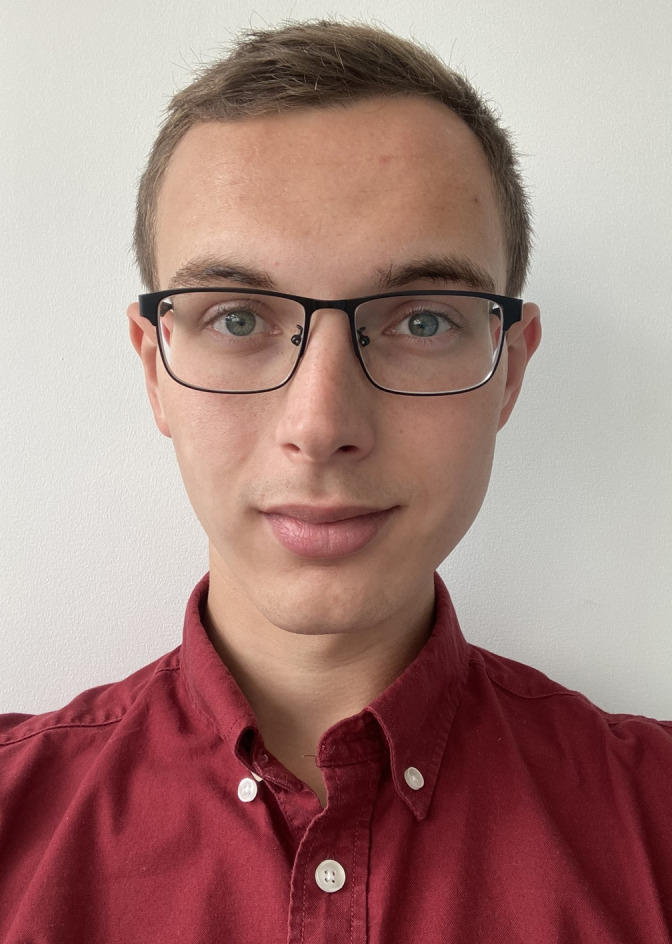

## Biographical Information

*Nicholas Measom completed his PhD in 2017 (GSK/University of Strathclyde Collaborative Industrial PhD programme, Dr. David Hirst and Dr. Craig Jamieson) working on the synthesis and application of bicyclo[1.1.1]pentanes within Lp‐PLA_2_ inhibitors. He joined GSK as a synthetic medicinal chemist in 2017 and became an Investigator in 2020*.

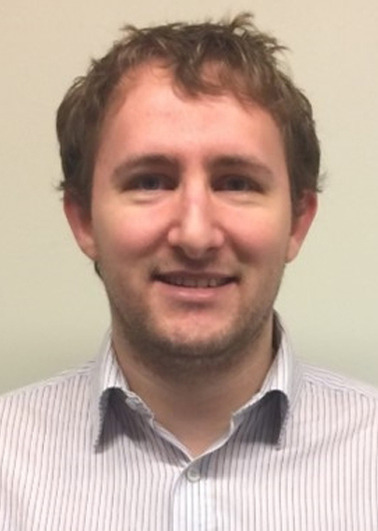

## Biographical Information

*John Murphy was born in Dublin and educated at the University of Dublin (TCD) and the University of Cambridge. After postdoctoral fellowships at Alberta and Oxford, he was appointed as Lecturer, then Reader, at the University of Nottingham. Since 1995, he has held the Merck‐Pauson Professorship at the University of Strathclyde. His interests are in reaction mechanisms and synthesis*.

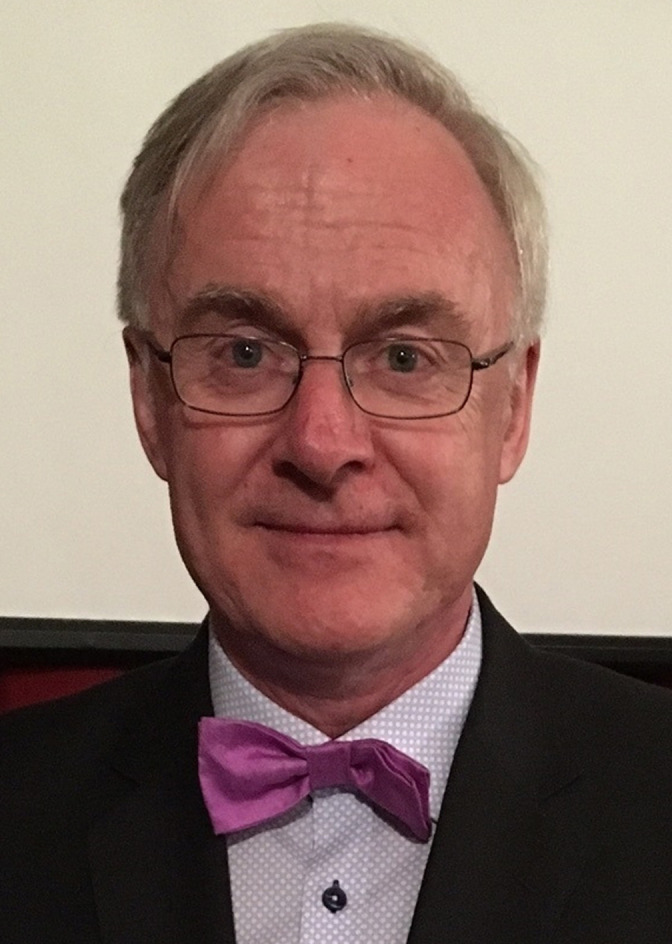

## Biographical Information

*Darren Poole completed his DPhil in 2014 (University of Oxford, Prof. Timothy Donohoe) where he worked on nucleophilic dearomatisation reactions, and hydrogen borrowing reactions of methanol. He joined GSK′s Flexible Discovery Unit as a synthetic chemist in 2014, and is now a Scientific Leader and GSK Fellow in the Medicinal Chemistry department. His research interests are particularly focussed on the application of new synthetic methodologies and technologies in drug discovery*.

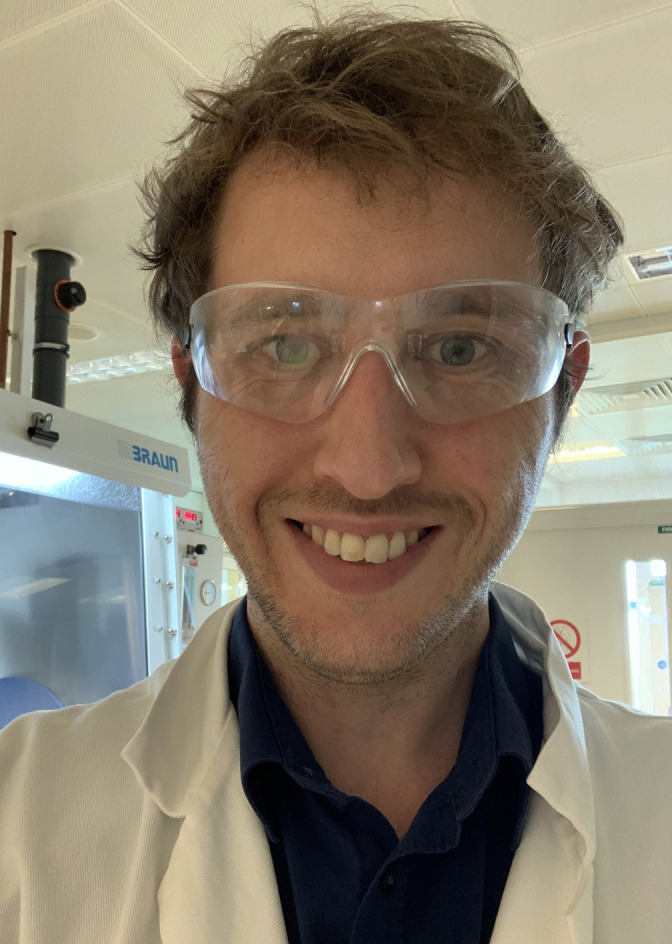

## References

[1] L. Itzhaki , E. Altus , H. Basch , S. Hoz , Angew. Chem. Int. Ed. 2005, 44, 7432–7435; 10.1002/anie.200502448 16240306

[2] P. I. Dron , K. Zhao , J. Kaleta , Y. Shen , J. Wen , R. K. Shoemaker , C. T. Rogers , J. Michl , Adv. Funct. Mater. 2016, 26, 5718–5732.

[3] P. F. H. Schwab , B. C. Noll , J. Michl , J. Org. Chem. 2002, 67, 5476–5485. 12153245 10.1021/jo020111b

[4] A. De Meijere , M. Messner , V. Vill , Mol. Cryst. Liq. Cryst. 1994, 257, 161–167.

[5] A. de Meijere , L. Zhao , V. N. Belov , M. Bossi , M. Noltemeyer , S. W. Hell , Chem. Eur. J. 2007, 13, 2503–2516. 17203493 10.1002/chem.200601316

[6] F. A. Cotton , L. M. Daniels , C. Lin , C. A. Murillo , S.-Y. Yu , J. Chem. Soc. Dalton Trans. 2001, 502–504.

[7] G. M. Locke , S. S. R. Bernhard , M. O. Senge , Chem. Eur. J. 2019, 25, 4590–4647. 30387906 10.1002/chem.201804225

[8] M. R. Barbachyn , D. K. Hutchinson , D. S. Toops , R. J. Reid , G. E. Zurenko , B. H. Yagi , R. D. Schaadt , J. W. Allison , Bioorg. Med. Chem. Lett. 1993, 3, 671–676.

[9] R. Pellicciari , M. Raimondo , M. Marinozzi , B. Natalini , G. Constantino , C. Thomsen , J. Med. Chem. 1996, 39, 2874–2876. 8709120 10.1021/jm960254o

[10] J. Kanazawa , M. Uchiyama , Synlett 2019, 30, 1–11.

[11] T. J. Ritchie , S. J. F. Macdonald , Drug Discovery Today 2009, 14, 1011–1020. 19729075 10.1016/j.drudis.2009.07.014

[12a] F. Lovering , J. Bikker , C. Humblet , J. Med. Chem. 2009, 52, 6752–6756; 19827778 10.1021/jm901241e

[12b] F. Lovering , MedChemComm 2013, 4, 515–519;

[12c] W. Wei , S. Cherukupalli , L. Jing , X. Liu , P. Zhan , Drug. Discovery Today 2020, 25, 1839–1845. 32712310 10.1016/j.drudis.2020.07.017

[13a] A. F. Stepan , I. V. E. Subramanyam , J. K. Dutra , T. J. O′Sullivan , K. J. DiRicio , W. S. McDonald , P. H. Dorff , C. E. Nolan , S. L. Becker , L. R. Pustilnik , D. R. Ridell , G. W. Kauffman , B. L. Kormos , L. Zhang , Y. Lu , S. H. Capetta , M. E. Green , K. Karki , E. Sibley , K. P. Atchinson , A. J. Hallgren , C. E. Oborski , A. E. Robshaw , B. Sneed , C. J. O'Donnell , J. Med. Chem. 2012, 55, 3414–3424; 22420884 10.1021/jm300094u

[13b] K. C. Nicolaou , D. Vourloumis , S. Totokotsopoulos , A. Papakyriakou , H. Karsunky , H. Fernando , J. Gavrilyuk , D. Webb , A. F. Stepan , ChemMedChem 2016, 11, 31–37; 26585829 10.1002/cmdc.201500510

[13c] Q. Pu , H. Zhang , L. Guo , M. Cheng , A. C. Doty , H. Ferguson , X. Fradera , C. A. Lesburg , M. A. McGowan , J. R. Miller , P. Geda , X. Song , K. Otte , N. Sciammetta , N. Solban , W. Yu , D. L. Sloman , H. Zhou , A. Lammens , L. Neumann , D. J. Bennett , A. Pasternak , Y. Han , ACS Med. Chem. Lett. 2020, 11, 1548–1554; 32832022 10.1021/acsmedchemlett.0c00195 PMC7429971

[13d] N. D. Measom , K. D. Down , D. J. Hirst , C. Jamieson , E. S. Manas , V. K. Patel , D. O. Somers , ACS Med. Chem. Lett. 2017, 8, 43–48; 28105273 10.1021/acsmedchemlett.6b00281 PMC5238484

[13e] E. G. Tse , S. D. Houston , C. M. Williams , G. P. Savage , L. M. Rendina , I. Hallyburton , M. Anderson , R. Sharma , G. S. Walker , R. S. Obach , M. H. Todd , J. Med. Chem. 2020, 63, 11585–11601; 32678591 10.1021/acs.jmedchem.0c00746

[13f] Y. P. Auberson , C. Brocklehurst , M. Furegati , T. C. Fessard , G. Koch , A. Decker , L. La Vecchia , E. Briard , ChemMedChem 2017, 12, 590–598. 28319646 10.1002/cmdc.201700082

[14] M. V. Westphal , B. T. Wolfstadter , J. M. Plancher , J. Gatfield , E. M. Carreira , ChemMedChem 2015, 10, 461–469. 25630804 10.1002/cmdc.201402502

[15] Y. L. Goh , Y. T. Cui , V. Pendharkar , V. A. Adsool , ACS Med. Chem. Lett. 2017, 8, 516–520. 28523103 10.1021/acsmedchemlett.7b00018 PMC5430408

[16] S. Gardner , A. Vinter , Innovative Pharm. Technol. 2009, 42–46.

[17a] M. D. Levin , P. Kaszynski , J. Michl , Chem. Rev. 2000, 100, 169–234; 11749237 10.1021/cr990094z

[17b] X. Ma , L. Nhat Pham , Asian J. Org. Chem. 2020, 9, 8–22;

[17c] E. W. Delia , I. J. Lochert , Org. Prep. Proced. Int. 1996, 28, 411–441;

[17d] M. O. Senge , N. Grover , Synthesis 2020, 52, 3295–3325.

[18] M. R. Bauer , P. Di Fruscia , S. C. C. Lucas , I. N. Michaelides , J. E. Nelson , R. I. Storer , B. C. Whitehurst , RSC Med. Chem. 2021, 12, 448–471. 33937776 10.1039/d0md00370k PMC8083977

[19] J. W. Scannell , J. Bosley , PLoS One 2016, 11, e0147215. 26863229 10.1371/journal.pone.0147215 PMC4749240

[20] R. W. Brown , Future Med. Chem. 2021, 13, 211–224. 33445971 10.4155/fmc-2020-0294

[21] K. B. Wiberg , Angew. Chem. Int. Ed. Engl. 1986, 25, 312–322;

[22] P. Kaszynski , A. C. Friedli , J. Michl , J. Am. Chem. Soc. 1992, 114, 601–620.

[23] K. B. Wiberg , Tetrahedron Lett. 1985, 26, 599–602.

[24] R. M. Jarret , L. Cusumano , Tetrahedron Lett. 1990, 31, 171–174.

[25a] M. Pecul , H. Dodziuk , M. Jaszuński , O. Lukin , J. Leszczyński , Phys. Chem. Chem. Phys. 2001, 3, 1986–1991;

[25b] M. D. Levin , S. J. Hamrock , P. Kaszynski , A. B. Shtarev , G. A. Levina , B. C. Noll , M. E. Ashley , R. Newmark , G. G. I. Moore , J. Michl , J. Am. Chem. Soc. 1997, 119, 12750–12761.

[26] K. Kovačević , Z. B. Maksić , J. Org. Chem. 1974, 39, 539–545.

[27] B. Maillard , J. C. Walton , J. Chem. Soc. Chem. Commun. 1983, 900–901.

[28] J. R. Bews , C. Glidewell , J. C. Walton , J. Chem. Soc. Perkin Trans. 2 1982, 1147–1453.

[29] K. B. Wiberg , V. Z. Williams , J. Org. Chem. 1970, 35, 369–373.

[30] R. E. Robinson , J. Michl , J. Org. Chem. 1989, 54, 2051–2053.

[31a] R. M. Bychek , V. Hutskalova , Y. P. Bas , O. A. Zaporozhets , S. Zozulya , V. V. Levterov , P. K. Mykhailiuk , J. Org. Chem. 2019, 84, 15106–15117; 31553875 10.1021/acs.joc.9b01947

[31b] J.-X. Zhao , Y. Chang , J. Elleraas , T. Montgomery , J. E. Spangler , S. K. Nair , M. Del Bel , G. M. Gallego , J. J. Mousseau , M. A. Perry , M. R. Collins , J. C. Vantourout , P. S. Baran , ChemRxiv 2020, 10.26434/chemrxiv.13120283.v1.

[32] M.-S. Lee , D. A. Hrovat , W. T. Borden , J. Am. Chem. Soc. 1995, 117, 10353–10357.

[33a] A. Padwa , E. Alexander , J. Am. Chem. Soc. 1967, 89, 6376–6377;

[33b] A. Padwa , E. Alexander , J. Am. Chem. Soc. 1968, 90, 6871–6873;

[33c] A. Padwa , E. Shefter , E. Alexander , J. Am. Chem. Soc. 1968, 90, 3717–3721;

[33d] A. Padwa , E. Alexander , M. Niemcyzk , J. Am. Chem. Soc. 1969, 91, 456–462.

[34] N. C. Yang , D.-D. H. Yang , J. Am. Chem. Soc. 1958, 80, 2913–2914.

[35a] E. C. Alexander , J. A. Uliana , J. Am. Chem. Soc. 1974, 96, 5644–5646;

[35b] K. B. Wiberg , B. S. Ross , J. J. Isbell , N. McMurdie , J. Org. Chem. 1993, 58, 1372–1376;

[35c] S. Iwasaki , Helv. Chim. Acta 1978, 61, 2831–2842.

[36a] A. Padwa , E. Alexander , J. Am. Chem. Soc. 1970, 92, 5674–5681;

[36b] A. Padwa , W. Eisenberg , J. Am. Chem. Soc. 1972, 94, 5852–5858. 5053863 10.1021/ja00771a052

[37] C. H. DePuy , Acc. Chem. Res. 1968, 1, 33–41.

[38] K. B. Wiberg , R. A. Fenoglio , V. Z. Williams , R. W. Ubersax , J. Am. Chem. Soc. 1970, 92, 568–571.

[39] A. Padwa , E. Alexander , J. Am. Chem. Soc. 1970, 92, 1796–1797.

[40] G. A. Olah , G. K. S. Prakash , G. Liang , J. Am. Chem. Soc. 1979, 101, 3932–3934.

[41] P. Dowd , Y. H. Paik , Tetrahedron Lett. 1986, 27, 2813–2816.

[42] M. B. Sponsler , D. A. Dougherty , J. Org. Chem. 1984, 49, 4978–4984.

[43a] H. K. Hall , C. D. Smith , E. P. Blanchard , S. C. Cherkofsky , J. B. Sieja , J. Am. Chem. Soc. 1971, 93, 121–130;

[43b] J. Kaleta , I. Rončević , I. Císařová , M. Dračínský , V. Šolínová , V. Kašička , J. Michl , J. Org. Chem. 2019, 84, 2448–2461. 30675778 10.1021/acs.joc.8b02780

[44] K. B. Wiberg , D. S. Connor , J. Am. Chem. Soc. 1966, 88, 4437–4441.

[45] E. W. Della , P. E. Pigou , C. H. Schiesser , D. K. Taylor , J. Org. Chem. 1991, 56, 4659–4664.

[46] S. J. Hamrock , J. Michl , J. Org. Chem. 1992, 57, 5027–5031.

[47] P. M. Warner , R. C. LaRose , R. F. Palmer , C.-M. Lee , D. O. Ross , J. C. Clardy , J. Am. Chem. Soc. 1975, 97, 5507–5512.

[48] A. B. Shtarev , E. Pinkhassik , M. D. Levin , I. Stibor , J. Michl , J. Am. Chem. Soc. 2001, 123, 3484–3492. 11472120 10.1021/ja0000495

[49] Y. Takahira , M. Chen , Y. Kawamata , P. Mykhailiuk , H. Nakamura , B. K. Peters , S. H. Reisberg , C. Li , L. Chen , T. Hoshikawa , T. Shibuguchi , P. S. Baran , Synlett 2019, 30, 1178–1182. 33767531 10.1055/s-0037-1611737 PMC7990259

[50] Q. Zhu , D. Ji , T. Liang , X. Wang , Y. Xu , Org. Lett. 2015, 17, 3798–3801. 26172446 10.1021/acs.orglett.5b01774

[51] M. Larrosa , S. Heiles , J. Becker , B. Spengler , R. Hrdina , Adv. Synth. Catal. 2016, 358, 2163–2171.

[52] X. Ma , D. L. Sloman , Y. Han , D. J. Bennett , Org. Lett. 2019, 21, 7199–7203. 31294572 10.1021/acs.orglett.9b02026

[53] R. McNamee , M. M. Haugland , J. Nugent , R. Chan , K. Christensen , E. A. Anderson , Chem. Sci. 2021, 12, 7480–7485. 34163838 10.1039/d1sc01836a PMC8171340

[54] D. E. Applequist , T. L. Renken , J. W. Wheeler , J. Org. Chem. 1982, 47, 4985–4995.

[55] X. Ma , W. Pinto , L. N. Pham , D. L. Sloman , Y. Han , Eur. J. Org. Chem. 2020, 4581–4605.

[56a] W. v. E. Doering , J. F. J. Coburn , Tetrahedron Lett. 1965, 15, 991–995;

[56b] K. B. Wiberg , G. M. Lampman , R. P. Ciula , D. S. Connor , P. Schertler , J. Lavanish , Tetrahedron 1965, 21, 2749–2769.

[57] P. Wipf , C. R. J. Stephenson , K. Okumura , J. Am. Chem. Soc. 2003, 125, 14694–14695. 14640630 10.1021/ja038623a

[58a] J. E. Jackson , G. B. Mock , M. L. Tetef , G. Zheng , M. Jones, Jr. , Tetrahedron 1985, 41, 1453–1464;

[58b] G. B. Mock , M. Jones, Jr. , Tetrahedron Lett. 1981, 22, 3819–3822.

[59a] E. C. Alexander , J. Uliana , J. Am. Chem. Soc. 1976, 98, 4324–4325;

[59b] E. C. Alexander , T. Tom , Tetrahedron Lett. 1978, 19, 1741–1742.

[60] C. Yang , W. Xia , J. R. Scheffer , M. Botoshansky , M. Kaftory , Angew. Chem. Int. Ed. 2005, 44, 5087–5089; 10.1002/anie.200500983 16035013

[61] M. Ma , C. Yang , B. Li , Y. Shao , G. Zhao , W. Xia , Chin. J. Chem. 2012, 30, 91–95.

[62] A. J. Sterling , A. B. Dürr , R. C. Smith , E. A. Anderson , F. Duarte , Chem. Sci. 2020, 11, 4895–4903. 34122945 10.1039/d0sc01386b PMC8159217

[63] S. Yu , A. Noble , R. B. Bedford , V. K. Aggarwal , J. Am. Chem. Soc. 2019, 141, 20325–20334. 31786925 10.1021/jacs.9b10689

[64a] K. Semmler , G. Szeimies , J. Belzner , J. Am. Chem. Soc. 1985, 107, 6410–6411;

[64b] K. Opitz , A. D. Schlüter , Angew. Chem. Int. Ed. Engl. 1989, 28, 456–458;

[64c] H. Bothe , A. D. Schlüter , Chem. Ber. 1991, 124, 587–590;

[64d] R. Freudenberger , W. Lamer , A.-D. Schlüter , J. Org. Chem. 1993, 58, 6497–6498;

[64e] R. Klopsch , A.-D. Schlüter , Tetrahedron 1995, 51, 10491–10496;

[64f] B. Stulgies , D. P. Pigg , P. Kaszynski , Z. H. Kudzin , Tetrahedron 2005, 61, 89–95;

[64g] C. Mazal , O. Škarka , J. Kaleta , J. Michl , Org. Lett. 2006, 8, 749–752; 16468758 10.1021/ol053039n

[64h] J. Kaleta , J. Michl , C. Mazal , J. Org. Chem. 2010, 75, 2350–2356. 20205462 10.1021/jo100169b

[65a] J. Belzner , G. Szeimies , Tetrahedron Lett. 1987, 28, 3099–3102;

[65b] G. Kottirsch , K. Polborn , G. Szeimies , J. Am. Chem. Soc. 1988, 110, 5588–5590;

[65c] J. Belzner , B. Gareiß , K. Polborn , W. Schmid , K. Semmler , G. Szeimies , Chem. Ber. 1989, 122, 1509–1529;

[65d] M. Kenndoff , A. Singer , G. Szeimies , J. Prakt. Chem. 1997, 339, 217–232.

[66] H. Hong , E. Zhang , J. Lu , F. Wei , S. Yang , G. Che (Jinlin Asymchem Laboratories Co., Ltd.), Continuous Synthesis Method for 1,1′-Bicyclo[1.1.1]pentane-1,3-diethyl Ketone Organic Matter, WO/2020/248126, 2020.

[67] A. M. Dilmaç , E. Spuling , A. de Meijere , S. Bräse , Angew. Chem. Int. Ed. 2017, 56, 5684–5718; 10.1002/anie.201603951 27905166

[68] X. Ma , Y. Han , D. J. Bennett , Org. Lett. 2020, 22, 9133–9138. 33170018 10.1021/acs.orglett.0c03612

[69] R. Gianatassio , J. M. Lopchuk , J. Wang , C.-M. Pan , L. R. Malins , L. Prieto , T. A. Brandt , M. R. Collins , G. M. Gallego , N. W. Sach , J. E. Spangler , H. Zhu , J. Zhu , P. S. Baran , Science 2016, 351, 241–246. 26816372 10.1126/science.aad6252 PMC4730898

[70] J. M. E. Hughes , D. A. Scarlata , A. C. Chen , J. D. Burch , J. L. Gleason , Org. Lett. 2019, 21, 6800–6804. 31407916 10.1021/acs.orglett.9b02426

[71] S. Yu , C. Jing , A. Noble , V. K. Aggarwal , Angew. Chem. Int. Ed. 2020, 59, 3917–3921; 10.1002/anie.201914875 31912941

[72] D. F. J. Caputo , C. Arroniz , A. B. Dürr , J. J. Mousseau , A. F. Stepan , S. J. Mansfield , E. A. Anderson , Chem. Sci. 2018, 9, 5295–5300. 29997886 10.1039/c8sc01355a PMC6001403

[73] Y. Yang , J. Tsien , J. M. E. Hughes , B. K. Peters , R. R. Merchant , T. Qin , ChemRxiv 2021, 10.26434/chemrxiv.13724827.v1.

[74] K. B. Wiberg , D. S. Connor , G. M. Lampman , Tetrahedron Lett. 1964, 5, 531–534.

[75] V. Fasano , A. W. McFord , C. P. Butts , B. S. L. Collins , N. Fey , R. W. Alder , V. K. Aggarwal , Angew. Chem. Int. Ed. 2020, 59, 22403–22407; 10.1002/anie.202007776 32866342

[76] D. S. Matteson , D. Majumdar , J. Am. Chem. Soc. 1980, 102, 7588–7590.

[77a] J. Kanazawa , K. Maeda , M. Uchiyama , J. Am. Chem. Soc. 2017, 139, 17791–17794; 29131599 10.1021/jacs.7b11865

[77b] X. Zhang , R. T. Smith , C. Le , S. J. McCarver , B. T. Shireman , N. I. Carruthers , D. W. C. MacMillan , Nature 2020, 580, 220–226; 32066140 10.1038/s41586-020-2060-z PMC7148169

[77c] J. H. Kim , A. Ruffoni , Y. S. S. Al-Faiyz , N. S. Sheikh , D. Leonori , Angew. Chem. Int. Ed. 2020, 59, 8225–8231; 10.1002/anie.202000140 PMC7318212 32003916

[78a] S. Murarka , Adv. Synth. Catal. 2018, 360, 1735–1753;

[78b] J. Xuan , Z.-G. Zhang , W.-J. Xiao , Angew. Chem. Int. Ed. 2015, 54, 15632–15641; 10.1002/anie.201505731 26509837

[78c] Y. Jin , H. Fu , Asian J. Org. Chem. 2017, 6, 368–385;

[78d] P. Niu , J. Li , Y. Zhang , C. Huo , Eur. J. Org. Chem. 2020, 5801–5814;

[78e] H. Chen , Y. A. Liu , X. Liao , Synthesis 2021, 53, 1–29.

[79a] L. Marzo , S. K. Pagire , O. Reiser , B. König , Angew. Chem. Int. Ed. 2018, 57, 10034–10072; 10.1002/anie.201709766 29457971

[79b] J. Twilton , C. Le , P. Zhang , M. H. Shaw , R. W. Evans , D. W. C. MacMillan , Nat. Rev. Chem. 2017, 1, 0052.

[80] A. W. Hung , A. Ramek , Y. Wang , T. Kaya , J. A. Wilson , P. A. Clemons , D. W. Young , Proc. Natl. Acad. Sci. USA 2011, 108, 6799–6804. 21482811 10.1073/pnas.1015271108 PMC3084099

